# One size Fitts’ all? Reconsidering the use of deviations from real-time action imagery in mental chronometry tasks

**DOI:** 10.3758/s13428-026-03124-8

**Published:** 2026-07-30

**Authors:** Carla Czilczer, Pierre Sachse, Stephan F. Dahm

**Affiliations:** University of Innsbruck, Faculty of Psychology and Sports Science, Department of Psychology, Universitätsstraße 5-7, 6020 Innsbruck, Austria

**Keywords:** Action imagery ability, Motor imagery, Fitts’ law, Mental chronometry, Tool use

## Abstract

Mental chronometry tasks commonly presume that real-time action imagery indicates high action imagery ability (AIA). However, factors that systematically influence imagery durations undermine the validity of AIA scores based on deviations from real-time action imagery. Therefore, we introduce and validate a novel approach for measuring AIA using mental chronometry. This approach relies on the relationship between a movement’s difficulty and its duration (Fitts’ law), with AIA scores quantifying the degree to which this relationship is preserved in action imagery. In a Chronometric Radial Fitts’ Task (CRFT), participants (*N* = 30) executed and imagined both tapping (with a stylus) and clicking (with a computer mouse) radially arranged targets. Single execution and imagery durations were isolated through simultaneous space bar presses. Targets varied in difficulty according to Fitts’ law, which held across all four conditions (for both tools, when executing and imagining the movements). The AIA scores derived from the proposed Fitts’ law approach outperformed those derived from earlier mental chronometry approaches, being the only scores indicating convergent validity across tools. Using the stylus, larger absolute and relative deviations from real-time action imagery were consistently associated with higher subjective AIA, reinforcing validity concerns. Participants struggled to incorporate the computer mouse’s properties into the internal model of the movement, resulting in imagery with the computer mouse relying on internal models similar to the stylus (simpler tool). In addition to offering a refined approach for measuring manipulation- and timing-related AIA, our study highlights the importance of tool familiarity in mental chronometry.

**Supplementary Information:**

The online version contains supplementary material available at https://doi.org/10.3758/s13428-026-03124-8.

## Introduction

The precise measurement of an individual’s ability to mentally simulate an action without executing it (*action imagery*; Decety et al., 1989; Jeannerod, 1994) is crucial for effectively applying action imagery practice to (re)learn and refine motor actions. Systematic action imagery practice has been shown to aid motor rehabilitation following stroke (López et al., 2019) and improve performance in professional sports (Simonsmeier et al., 2021). Yet, an individual’s action imagery ability may determine the effects of action imagery practice (Robin et al., 2007). Individuals that possess high action imagery ability (“good imagers”) are thought to exhibit neural activation patterns that closely resemble those observed during action execution (Holmes & Collins, 2001). Accurate action imagery is assumed to strengthen neural networks involved in action execution and thus benefit action execution (Cumming & Williams, 2012; Holmes & Collins, 2001). In other words, the accuracy with which action imagery resembles action execution delineates poor and good imagers, and determines its effects. Precise measures of action imagery ability that are sensitive to interindividual differences are therefore important to advance research on action imagery and its effective application in motor rehabilitation and sports.

Measuring action imagery ability presents significant challenges due to its complexity and lack of direct observability (Dahm, 2020). Action imagery (also referred to as motor imagery, movement imagery, or action simulation; Moreno-Verdú et al., 2024) encompasses multiple sensory modalities, most notably visual and kinesthetic sensations (Krüger et al., 2022). In visual action imagery, the imager can take a first-person (internal) or third-person (external) perspective. Moreover, imagining an action involves generating a vivid mental representation, and maintaining and manipulating it (Kosslyn et al., 2006; Kraeutner et al., 2020). Given these multiple modalities and cognitive demands, action imagery ability can be conceptualized as a conglomerate of interrelated components. Various approaches exist to measure one or multiple components of action imagery ability.

*Subjective measures* such as self-report questionnaires (e.g., Roberts et al., 2008; Watt, 2003; Williams et al., 2012) are frequently used to assess visual and/or kinesthetic action imagery ability (e.g., Mandolesi et al., 2024; Wright et al., 2024). Their advantage lies in their practicality and ease of administration. However, self-report questionnaires are susceptible to bias (e.g., avoidance of negative self-image: Alicke et al., 1995; Alicke & Sedikides, 2009; social desirability: Nederhof, 1985), which can obscure genuine interindividual differences. Self-report questionnaires capture phenomenological aspects of imagery, such as vividness (Roberts et al., 2008) and ease of imagery generation (Williams et al., 2012). When rating these aspects, respondents may not have experienced the full range of imagery quality defined by the scale, so rating points (e.g., Roberts et al., 2008: “clear and reasonably vivid”) can be interpreted differently across individuals (Schwarzkopf, 2024). This may contribute to weak and inconsistent correlations between subjective and objective measures (e.g., Dahm et al., 2022; Kraeutner et al., 2020; Moreno-Verdú et al., 2026; Muraki & Pexman, 2021; Suggate, 2024; Williams et al., 2015), which also reflect that objective measures index the strength, accuracy, or efficiency of action imagery rather than its phenomenology. Subjective measures may therefore be particularly informative for understanding intraindividual variation in imagery experience, whereas objective measures are better suited for quantifying interindividual differences in the functional use of action imagery.

*Mental chronometry tasks* compare durations of executing and imagining specific movements to provide an objective and practical measure of action imagery ability, with smaller deviations typically indicating higher ability (Beauchet et al., 2010; Decety et al., 1989). Mental chronometry tasks are more sensitive to interindividual differences than subjective measures and other behavioral tasks, which show ceiling effects (e.g., Dahm et al., 2019; Madan & Singhal, 2013). A key limitation, however, lies in the assumption that real-time action imagery inherently reflects high action imagery ability. Imagery durations vary systematically with individual, environmental, and task-related factors (Guillot & Collet, 2005; Guillot et al., 2012), which may be independent of underlying ability (Reed, 2002). We therefore propose an adapted mental chronometry approach that accounts for such influencing factors and builds on theoretical (Rieger et al., 2024) and empirical work demonstrating shared mechanisms between action execution and action imagery (e.g., Fitts, 1954). We evaluate the validity of this approach against mental chronometry approaches relying on deviations from real-time action imagery.

### The assumption of real-time action imagery

The assumption that real-time action imagery reflects accurate imagery is based on *functional similarities* between action execution and action imagery (Jeannerod, 2001). In both, intending an action comes with intending certain action effects (Bach et al., 2022). Alongside the given affordances, inverse models select motor commands to realize the intended effects (Rieger et al., 2024). Forward models then use efference copies to predict the action effects, allowing fine-tuning by inverse models when discrepancies arise between intended and predicted effects (Blakemore et al., 2002; Davidson & Wolpert, 2005). Action execution and action imagery coincide in this movement planning phase (Glover & Baran, 2017). Supporting evidence includes research on physiological parameters (Kilteni et al., 2018; Oishi et al., 2000) and neural activation showing overlapping, though partly distinct, brain areas involved in action execution and action imagery (Hardwick et al., 2018; Hétu et al., 2013; Munzert et al., 2009; Van Caenegem et al., 2026). Although performance in mental chronometry tasks could in principle rely on tacit or propositional knowledge about movement durations rather than imagery per se (Annett, 1996; Pylyshyn, 2002), durations of executed and imagined movements obey the same psychophysical (e.g., Fitts’ law: Decety & Jeannerod, 1995; Fitts, 1954) and biomechanical principles (e.g., the two-thirds power law; Papaxanthis et al., 2012; Viviani & Terzuolo, 1982). This is consistent with the view that action imagery involves similar mechanisms as action execution (Rieger et al., 2024).

Besides their similarities, there are also differences between action execution and action imagery, particularly in the movement realization phase (Glover & Baran, 2017). Whilst sensory feedback informs the actor in action execution (Wolpert & Ghahramani, 2000), conscious elaboration relying on executive functions is involved in action imagery and thus requires more cognitive resources (Glover & Baran, 2017). In mental chronometry tasks, participants indicate the start and end of an imagined movement (e.g., tapping a start/stop-box: Caeyenberghs et al., 2009a). Such actual responses during action imagery require an attentional shift when switching between action execution and action imagery (Dahm & Rieger, 2016a; Rieger et al., 2017). According to the motor-cognitive model (Glover & Baran, 2017), this shift becomes more challenging when the mental representation of the action lacks precision and detail (low *fidelity*), as more executive resources are needed. Depleting executive resources (e.g., due to a low-fidelity mental representation) is assumed to result in longer imagery durations, while timing accuracy improves with the fidelity of the mental representation. Real-time action imagery may therefore indicate a high-fidelity mental representation of an action, which in turn requires a high level of action imagery ability.

### The duration of action imagery

When participants are instructed to engage in real-time action imagery, discrepancies between execution and imagery durations may indicate difficulties in accurately simulating the intended action (Glover & Baran, 2017; Martel & Glover, 2023). In such circumstances, the *absolute deviation* from real-time action imagery quantifies the underlying action imagery ability (see Eq. 1; AIA score = action imagery ability score; $\overline{\mathrm{M}\mathrm{T} }$ = mean movement time; AE = action execution; AI = action imagery).

1 $$\mathrm{A}\mathrm{I}\mathrm{A}\text{ }\mathrm{s}\mathrm{c}\mathrm{o}\mathrm{r}\mathrm{e}= {\overline{\mathrm{M}\mathrm{T}} }_{\mathrm{A}\mathrm{E}}-{\overline{\mathrm{M}\mathrm{T}} }_{\mathrm{A}\mathrm{I}}$$

However, deviations from real-time action imagery may stem from other factors that are distinct from action imagery ability, including cognitive fatigue (Podda et al., 2020), physical fatigue (Di Rienzo et al., 2012), and arousal level (Louis et al., 2011). Further systematic deviations from real-time action imagery are attributable to the characteristics of the task (Guillot & Collet, 2005; Guillot et al., 2012). Imagery durations tend to be longer than execution durations for shorter tasks and shorter than execution durations for longer tasks (Dahm & Rieger, 2016b; Debarnot et al., 2012; Grealy & Shearer, 2008; Orliaguet & Coello, 1998). Moreover, imagery durations were shown to exceed execution durations in more complex tasks (Calmels & Fournier, 2001; Reed, 2002), fitting their higher load on executive resources (Glover & Baran, 2017; Reed, 2002).

In a study with springboard divers (Reed, 2002), intermediates tended to imagine complex dives more slowly than experts and novices, resulting in the largest deviations from real-time action imagery. This was attributed to intermediates’ non-automatized knowledge of the complex dive. Experts may rely on more automatized mental representations, whereas novices may lack the knowledge needed to mentally represent the dive’s complexity. Intermediates, in contrast, may consciously elaborate on the imagined action, thereby prolonging its duration. This illustrates how intertwined factors from different sources (here: task-related, individual) can lead to systematic deviations from real-time action imagery, and thus intermediates scoring worse than novices. More broadly, the multitude of individual, environmental, and task-related factors influencing imagery durations undermines the isolation of action imagery ability as the sole determinant of real-time action imagery. Therefore, using absolute deviations from real-time action imagery as a direct indicator of an individual’s action imagery ability is questionable.

### Limitations of absolute and relative deviations in mental chronometry

To account for systematic influences on absolute durations in mental chronometry, *relative deviations* were used to measure an individual’s action imagery ability. This typically involves calculating the difference between execution and imagery durations divided by execution durations (see Eq. 2; Lambert et al., 2024).

2 $$\mathrm{A}\mathrm{I}\mathrm{A} \text{ }\mathrm{s}\mathrm{c}\mathrm{o}\mathrm{r}\mathrm{e}=\frac{{\overline{\mathrm{M}\mathrm{T}} }_{\mathrm{A}\mathrm{E}}-{\overline{\mathrm{M}\mathrm{T}} }_{\mathrm{A}\mathrm{I}}}{{\overline{\mathrm{M}\mathrm{T}} }_{\mathrm{A}\mathrm{E}}}$$

Using relative instead of absolute deviations is advantageous when some individuals’ execution and imagery durations are influenced by specific factors in the exact same way. Suppose two individuals, slow Suzie and fast Felix, execute and imagine the same action (e.g., tapping targets). Suppose that slow Suzie has more difficulty in maintaining precision (i.e., hitting the targets) than fast Felix. Slow Suzie executes the task in 10 s and imagines it in 12.5 s (+ 25%), while fast Felix executes it in 6 s and imagines it in 7.5 s (+ 25%). If absolute deviations were used, slow Suzie would appear to have poorer action imagery ability (+ 2.5 s) than fast Felix (+ 1.5 s), despite the fact that her mental representation accurately incorporated the difficulty that she encountered in precisely tapping the targets. Conversely, if fast Felix imagined the task in 8.5 s instead of 7.5 s, absolute deviations would classify both as equally accurate (+ 2.5 s), even though his imagery duration would then deviate by 42%.

Applying relative instead of absolute deviations in mental chronometry relies on two key assumptions: (1) Action execution and action imagery are equally affected by influencing factors, and (2) imagery duration scales proportionally with execution duration. For instance, longer imagery durations place greater demands on cognitive resources, increasing the likelihood of accumulating small errors. Hence, relative deviations can account for scenarios in which individuals execute and imagine a task more slowly than others due to the same underlying factor (here: greater difficulty in executing the task).

However, the frequent occurrence of systematic discrepancies between execution and imagery durations (Guillot & Collet, 2005; Guillot et al., 2012) suggests that action execution and action imagery are not always equally influenced by individual, environmental, and task-related factors. A particular challenge arises when such factors selectively affect imagery durations only. Task difficulty and expertise provide clear examples of this selective influence. Although experts are more likely to imagine complex movements in real time (Reed, 2002), they still tend to adjust action imagery speed in response to perceived difficulty (Calmels & Fournier, 2001; Calmels et al., 2006). In the aforementioned study of springboard divers (Reed, 2002), intermediates showed greater deviations from real-time action imagery than novice divers, despite likely having a more refined mental representation of the dive. In this case, task-related factors and expertise influenced action execution and action imagery differently, reflecting an asymmetry that relative deviations cannot account for.

### A novel mental chronometry approach

To account for systematic deviations from real-time action imagery, a *constraint approach* was proposed (Dahm, 2020). It approximates action imagery ability components via the proportional change in execution and imagery durations after introducing a constraint to the action. These proportional changes in execution and imagery durations are subtracted, such that smaller differences indicate higher action imagery ability (see Eq. 3; BL = baseline condition; C = constraint condition).

3 $$\mathrm{A}\mathrm{I}\mathrm{A} \text{ }\mathrm{s}\mathrm{c}\mathrm{o}\mathrm{r}\mathrm{e}=\frac{{\overline{\mathrm{M}\mathrm{T}} }_{\mathrm{A}\mathrm{E}, \mathrm{C}} -{\overline{\mathrm{M}\mathrm{T}} }_{\mathrm{A}\mathrm{E}, \mathrm{B}\mathrm{L}}}{{\overline{\mathrm{M}\mathrm{T}} }_{\mathrm{A}\mathrm{E}, \mathrm{B}\mathrm{L}}}- \frac{{\overline{\mathrm{M}\mathrm{T}} }_{\mathrm{A}\mathrm{I}, \mathrm{C}}-{\overline{\mathrm{M}\mathrm{T}} }_{\mathrm{A}\mathrm{I}, \mathrm{B}\mathrm{L}}}{{\overline{\mathrm{M}\mathrm{T}} }_{\mathrm{A}\mathrm{I}, \mathrm{B}\mathrm{L}}}$$

We extend this by incorporating Fitts’ law (1954), translating the constraint into an increase in movement difficulty to enhance the accuracy and robustness of the score. Fitts’ law states that movement time depends on the *movement difficulty* (often referred to as index of difficulty). To maintain accuracy, movements slow down for more difficult tasks, reflecting a speed–accuracy trade-off. This can be described by Eq. 4, with intercept *a* and slope *b* as the effect of movement difficulty on MT.

4 $$\mathrm{M}\mathrm{T}=a+b\times \mathrm{m}\mathrm{o}\mathrm{v}\mathrm{e}\mathrm{m}\mathrm{e}\mathrm{n}\mathrm{t} \text{ }\mathrm{d}\mathrm{i}\mathrm{f}\mathrm{f}\mathrm{i}\mathrm{c}\mathrm{u}\mathrm{l}\mathrm{t}\mathrm{y}$$

Fitts’ law is typically implemented in tasks where participants point to targets (e.g., with a pen) as quickly and accurately as possible. The movement difficulty is determined by the amplitude *A* to the target and the width *W* of the target (Eq. 5), such that movement times MT are prolonged for increasingly distant and small targets.

5 $$\text{movement\, difficulty}={\mathrm{log}}_2\left(\frac{2\mathrm{A}}{\mathrm{W}}\right)$$

Varying the movement difficulty enables a comparison of its effects on movement times (slope *b* in Eq. 4) in action execution and action imagery. Like relative deviations from real-time action imagery, the constraint approach accounts for interindividual differences in execution durations. Beyond that, it accounts for factors that influence imagery durations while leaving execution durations unchanged—provided their influence is consistent across movement difficulties. This is important for factors originating from the specific action or setting that do not reflect underlying action imagery ability. For instance, individuals are not penalized for slowing down to accurately imagine a more complex part of a movement (e.g., hitting a target with the tip of the pen; imagining the flight phase of a somersault: Calmels et al., 2006). Such slowing may reflect a more refined mental representation than an imagery that disregards the complexity of this movement part. Accurate timing across difficulty levels requires appropriate adjustments in speed, acceleration, and precision that mirror those required during action execution. Hence, the extent to which movement difficulty similarly constrains execution and imagery durations can provide a meaningful indicator of action imagery ability components.

### Fitts’ law in mental chronometry

Despite proposed adaptations to accommodate Fitts’ law for broader or more specific cases of application (see Plamondon & Alimi, 1997), a substantial body of research has demonstrated that Fitts’ law holds across a range of movements, populations, and conditions (Bai et al., 2025; Caeyenberghs et al., 2009b; Choudhury et al., 2007; Drury & Woolley, 1995; Fitts & Peterson, 1964; Huys et al., 2015; Roberts et al., 2025; Wu et al., 2010). Consistent with the assumption of functional similarities, Fitts’ law applies to both executed and imagined movements (Bakker et al., 2007; Caeyenberghs et al., 2009b; Cerritelli et al., 2000; Decety & Jeannerod, 1995; Maruff et al., 1999a). In pointing tasks with targets of varying width, compliance with Fitts’ law and the correlation between execution and imagery durations have been used to assess interindividual differences in action imagery ability (Caeyenberghs et al., 2009a, 2009b; Choudhury et al., 2007; Smits-Engelsman & Wilson, 2013).

Findings from such pointing tasks suggest that participants do not simply estimate movement durations based on visual stimuli (i.e., targets), but genuinely engage in action imagery. For instance, convergence between executed and imagined movements increases for older relative to younger children, as indicated by greater alignment of slopes *b* for action execution and action imagery (see Eq. 4; Caeyenberghs et al., 2009b), and greater compliance with Fitts’ law (Caeyenberghs et al., 2009a). This likely reflects cerebral maturation, accompanied by the refinement of internal models and, consequently, more accurate movement planning and action imagery. Furthermore, Fitts’ law was shown to be sensitive to deficits in action imagery ability, as children with developmental coordination disorder adhered to Fitts’ law during action execution but not during action imagery (Maruff et al., 1999b; Wilson et al., 2001). Based on these findings, we expected that healthy adults would adhere to Fitts’ law in both action execution and action imagery, provided they genuinely engaged in action imagery.

### The Chronometric Radial Fitts’ Task

In the following, we demonstrate how components of action imagery ability, related to accurate manipulation and timing, can be measured by combining Fitts’ law with mental chronometry (*Fitts’ law approach*). We adapted previous tasks involving pointing movements of varying difficulty (e.g., Caeyenberghs et al., 2009a; Choudhury et al., 2007; Ferguson et al., 2015; Sirigu et al., 1995) into the *Chronometric Radial Fitts’ Task* (CRFT). Because pointing tasks requiring continuous directional adjustments are particularly sensitive to deficits in action imagery ability (Ferguson et al., 2015), the CRFT is based on movements to radially arranged targets (e.g., Caeyenberghs et al., 2009a). Participants additionally press the space bar with each executed and imagined tap, thereby enabling precise measurement of execution and imagery durations. We evaluated the robustness of the Fitts’ law approach in the CRFT along two pillars: (1) by testing it with two different tools, and (2) by comparing the distribution of AIA scores and their convergent validity between tools for the Fitts’ law approach versus alternative mental chronometry approaches.

#### Testing the Fitts’ law approach with different tools

As a first pillar, we tested the Fitts’ law approach using two tools with distinct properties in the CRFT. Participants performed the “tapping” either with a stylus (similar to prior studies, Caeyenberghs et al., 2009a) or with a computer mouse (resembling measures in online settings).

##### Adherence to Fitts’ law across actions and tools

Pointing and imagining pointing to targets with novel tools has been shown to follow Fitts’ law (Macuga et al., 2012), as mental representations of a movement adapt to a tool’s physical properties (Jacobs et al., 2010; Martin et al., 2011). Still, to draw valid conclusions about action imagery ability for both tools, movement times must adhere to Fitts’ law in all four CRFT conditions: action execution and action imagery (*action conditions*) with the stylus and the computer mouse (*tool conditions*). If, during action imagery, Fitts’ law holds only when using the stylus but not the computer mouse, participants failed to form an appropriate internal model for using the computer mouse, making it unsuitable for the CRFT. Further, we examine the variability in the slopes *b*. A lack of variance would indicate that the CRFT, combined with the Fitts’ law approach, is not sensitive enough to detect interindividual differences in action imagery ability.

##### Tool-dependent differences in execution and imagery durations

Hitting targets with a computer mouse is expected to be more challenging than tapping targets with a stylus. A computer mouse contains more unfamiliar properties (e.g., speed, cursor acceleration, sensitivity) that need to be incorporated into internal models of movement. Therefore, we expected slower movement times when using a computer mouse, and changing target size (and thereby movement difficulty) to have a greater impact on movement times in the computer mouse conditions (indicated by steeper slopes *b*). Unlike during action execution, visual feedback is absent during action imagery (Dahm & Rieger, 2022). This may pose a particular challenge for imagining clicking the targets quickly and accurately (e.g., missing visual feedback on oversteering). Consequently, we expect larger absolute deviations between execution and imagery durations in the computer mouse than the stylus conditions.

Although closed, repetitive, and not overly complex movements such as those in the CRFT facilitate real-time action imagery (Guillot & Collet, 2005), systematic deviations from real-time action imagery arising from individual, environmental, and task-related factors are likely. In pointing tasks involving horizontal movements only, imagery durations have commonly been found to exceed execution durations (Cerritelli et al., 2000; Maruff et al., 1999b; Wilson et al., 2001), except in certain populations (children with developmental coordination disorder: Choudhury et al., 2007; adolescents compared to adults: Ferguson et al., 2015) or in tool use contexts (Macuga et al., 2012). In contrast, pointing tasks requiring directional adjustments show shorter imagery than execution durations (Caeyenberghs et al., 2009a, 2009b; Ferguson et al., 2015; Smits-Engelsman & Wilson, 2013). These inconsistencies do not permit a clear prediction as to whether imagery durations will be longer or shorter than execution durations. Still, significant deviations between execution and imagery durations could point toward the need for the Fitts’ law approach to account for systematic influences on imagery durations.

#### Testing the Fitts’ law approach against alternative mental chronometry approaches

As a second pillar, we compare the AIA scores derived from the Fitts’ law approach to those obtained from established and previously proposed approaches (i.e., absolute deviation, relative deviation, constraint approaches). We determine whether the use of different tools in the CRFT yields converging results on individuals’ action imagery ability, and whether these results depend on the mental chronometry approach applied. In doing so, we test whether the Fitts’ law approach outperforms the alternatives.

## Methods

### Participants

A Monte Carlo simulation of a linear mixed model (random intercept for participant; fixed effects) determined the required number of participants and observations (α =.05, 80% power). Although strong associations between movement difficulty and movement time have been observed (e.g., Wong et al., 2013), we selected a conservative moderate effect size for movement difficulty (β =.3) to ensure sufficient power for participants with low action imagery ability, where action imagery likely complies less with Fitts’ law (e.g., Wilson et al., 2001). Given the uncertainty regarding the size of the fixed effects of action condition, tool condition, movement difficulty × tool condition, and tool condition × action condition in this specific task and tool context, we conservatively selected small respective effect sizes (βs =.2). We additionally estimated the sample size required to detect meaningful convergent validity (Carlson & Herdman, 2012) between the AIA scores of the tool conditions (ρ ≈.5), acknowledging that measures approximating the exact same underlying construct would be expected to show higher convergence (ρ ≈.7). This resulted in a minimum sample size of 30 participants, with 15 observations per movement difficulty for each participant within each CRFT condition.

Undergraduate Psychology students from the University of Innsbruck participated in the experiment. Participation was voluntary and was compensated with course credits. Of 38 participants, eight participants were excluded as they failed the data quality checks. Six of the excluded participants were noncompliant with instructions (i.e., action execution in action imagery trials, using the finger instead of the stylus, responding to the wrong targets, missing space bar presses), one participant was unable to press the space bar simultaneously with each tap or click, and one participant repeated the comprehension checks seven times.

The final sample (*N* = 30) was predominantly female (20 female, 10 male) and on average 23.2 years old (*SD* = 2.2; range 19–28 years). Approximately 43% of the participants reported prior experience with action imagery (dichotomous single item), through either sports or previous experiments. All participants reported having consistently used the hand they would usually use for operating the computer mouse (29 right, 1 left) and the stylus (24 right, 6 left) in the respective tool conditions. All participants had normal or corrected-to-normal vision.

### Experimental setup

The experiment was conducted in a laboratory and took about 40 min. The setup included a 24″ touchscreen monitor (Philips 242B9TL/00, running Windows 10) placed perpendicular to the table, an active stylus (Hama Stylus Pro 00125113; 1.5 mm tip), an optical mouse (default speed setting of 10/20 in Windows), and a QWERTZ keyboard. The experiment was programmed using OpenSesame 4.0.5 (Mathôt et al., 2012) with OSWeb backend and hosted on JATOS (Lange et al., 2015). Instructions were displayed on-screen with minimum presentation time, after which participants advanced at their own pace.

### Procedure

**Fig. 1:**
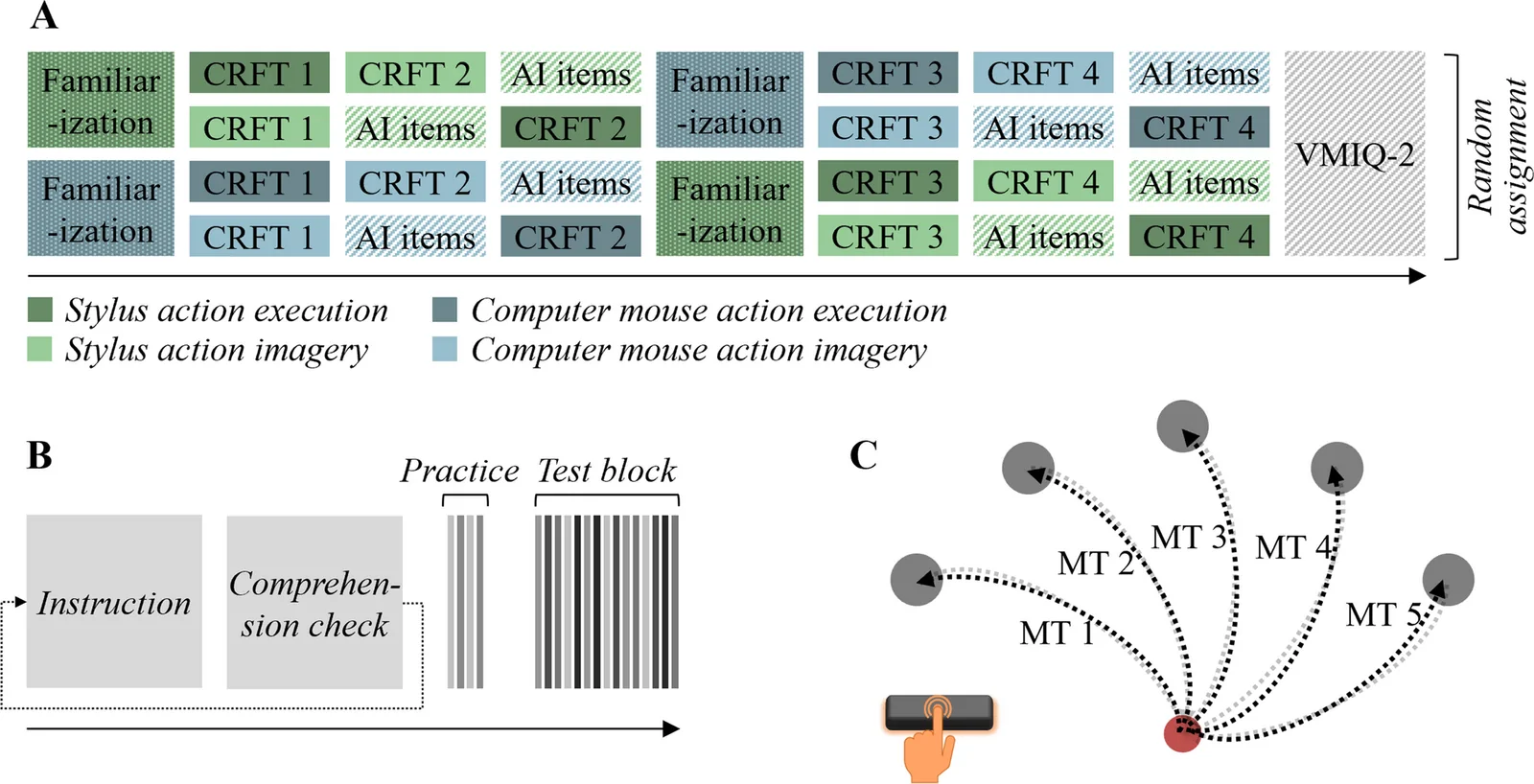
Experimental procedure, conditions, and trials in the Chronometric Radial Fitts’ Task (CRFT). **A** Overall experimental procedure. Participants were randomly assigned to one of four possible CRFT condition orders. Action imagery items (AI items) were tailored to the prior action imagery CRFT condition. **B** Procedure for each CRFT condition. Rectangles represent single trials, with different shades representing randomly ordered movement difficulties. **C** Movement sequence within a CRFT trial comprising five forward and five return movements between the red central circle and the gray targets (from left to right). Participants pressed the spacebar with their opposite hand upon tapping or clicking the central circle or a target. Movement times for forward movements (i.e., from the central circle to a target) were recorded, resulting in five observations per trial (MT 1 to 5)

The experiment had a 2 (action condition) × 2 (tool condition) within-subjects design. Participants completed four test blocks related to the four CRFT conditions: *stylus AE*, *stylus AI*, *computer mouse AE*, *computer mouse AI* (AE = action execution; AI = action imagery). Participants were randomly assigned to begin with either the two stylus conditions or the two computer mouse conditions. To control for order and carryover effects, it was randomized whether participants started with action execution or action imagery within each tool condition (see Fig. 1A). Before the first (when using a new tool) and third (when switching tools) CRFT condition, participants completed a *Familiarization Task*. Using the tool of the subsequent CRFT conditions (i.e., stylus or computer mouse), participants responded to 70 targets (width from 20 to 200 pixels) that appeared sequentially at different locations on the screen. Participants were instructed to tap or click them as fast and accurately (i.e., to actually hit the targets) as possible with the hand they usually used for tapping/clicking targets (hereafter referred to as dominant hand). Afterwards, participants received feedback on their accuracy in the Familiarization Task to endorse accurate aiming.

Participants then proceeded to the *CRFT* condition. Each CRFT condition started with reading instructions and completing a multiple-choice comprehension check (see Fig. 1B). Failing the comprehension check required rereading instructions and trying again until the correct response was given. Participants were then familiarized with the CRFT condition in a practice block of four trials with feedback on total trial duration (i.e., duration of the whole movement sequence). Afterwards, participants completed a test block of 15 trials without feedback. After the action imagery conditions of the CRFT, participants rated the quality of their imagery along nine *action imagery items*. After the last CRFT condition, participants completed the *Vividness of Movement Imagery Questionnaire 2* (VMIQ-2; Roberts et al., 2008).

### Measures

#### Chronometric Radial Fitts’ Task

The CRFT assessed components of participants’ action imagery ability related to manipulation and timing via mental chronometry. Based on earlier radial pointing tasks (e.g., Caeyenberghs et al., 2009a; Ferguson et al., 2015), we developed the CRFT to enhance the accuracy of movement time measurement and to avoid action execution immediately before action imagery. The same basic experimental setup was used across CRFT conditions (touchscreen monitor perpendicular to the table, keyboard), with the respective tool added in the action execution conditions (stylus or computer mouse).

Each trial comprised a full movement sequence. A response to a starting circle initiated the trial (action execution: tool response with the dominant hand; action imagery: spacebar press with the nondominant hand) to ensure the correct starting position. This response triggered a 3-s countdown, during which the central circle and the targets for that trial appeared. Participants were asked to start the movement sequence quickly after the countdown. The movement sequence began with a response to the central circle and continued with alternating movements between the central circle and five radially arranged targets from left to right (see Fig. 1C), such that the movement sequence started and ended at the central circle (after 11 responses). Participants were instructed to execute and imagine performing the movement sequence as fast and accurately (i.e., to actually hit the targets) as possible and not to correct their responses.

In the action execution conditions, participants responded to the central circle and targets by tapping (stylus) or clicking (computer mouse) them. These discrete responses (taps/clicks) ensured that participants deliberately aimed at each target. In the action imagery conditions, participants were instructed to focus on kinesthetic and internal visual sensations associated with tapping or clicking the central circle and targets while keeping their eyes open. In all CRFT conditions, participants were additionally instructed to press the spacebar simultaneously with each executed or imagined response using the index finger of the nondominant hand.

The action imagery instruction was intended to emphasize sensory aspects likely relevant for the required movements. Visual action imagery may support accurate taps and clicks, whereas kinesthetic action imagery may be crucial for adjusting acceleration, speed, and force. Research indicates that visual sensations are essential for goal-directed actions that require precision (Krüger et al., 2020). Imagining kinesthetic sensations may be crucial for acquiring accurate timing in action imagery (Féry, 2003), given that kinesthetic sensations vary with movement speed during action execution (Smith & Wakefield, 2013).

Across trials, the location and width of the central circle (width = 80 px) and the distance to the targets (practice block: amplitude = 400 px; test block: amplitude = 458 px) were fixed. The width of the targets varied across trials (practice block = 160 px, 40 px; test block = 224 px, 112 px, 56 px, 28 px, 14 px) to predict a linear increase in movement times according to Fitts’ law (Eq. 4). Because only the forward movements, not the return movements, varied in movement difficulty (practice block: easy = 2.32 and medium = 4.32; test block: 2.03, 3.03, 4.03, 5.03, 6.03), movement times were isolated to forward movements within the movement sequence. The simultaneous spacebar presses made this isolation of movement times possible and also ensured identical measurement across CRFT conditions. Thus, each trial yielded five observations of the duration of executing or imagining a movement from the central circle to a target, indicated by spacebar presses. Practice blocks comprised four trials (two movement difficulties, each presented twice) and test blocks comprised 15 trials (five movement difficulties, each presented three times) in random order. Per participant, this resulted in 15 movement time observations per movement difficulty and CRFT condition, 75 per CRFT condition, and 300 in total.

#### Subjective action imagery quality

We assessed participants’ subjective CRFT-specific and general action imagery quality to explore whether subjective action imagery quality was associated with objective action imagery ability in the CRFT and whether this depended on the mental chronometry approach.

##### Action imagery items

Nine action imagery items assessed the subjective quality and involvement of sensory modalities when imagining performing the movements in the CRFT for each tool condition. Participants rated (from 0 to 10) the *ease* and *speed* of generating action imagery, as well as action imagery *maintenance*, *control*, and *clarity*, and the *visual*, *kinesthetic*, *tactile*, and *auditory* sensations (e.g., “How well could you maintain imagery of the action?”; “How well could you see the action in imagery?”) during action imagery in the respective CRFT conditions. These items were adapted from the Sport Imagery Ability Measure (Watt, 2003).

##### Vividness of Movement Imagery Questionnaire 2

The German version (Dahm et al., 2019) of the *Vividness of Movement Imagery Questionnaire 2* (VMIQ-2; Roberts et al., 2008) assessed participants’ general subjective quality of action imagery. Participants rated the vividness of their internal visual (*M* ± *SD* = 4.1 ± 0.9; Cronbach’s α =.93), external visual (*M* ± *SD* = 3.8 ± 1.0; α =.92), and kinesthetic action imagery (*M* ± *SD* = 4.0 ± 1.0; α =.89) across 12 everyday actions on a five-point rating scale (1 = *no image at all, I only know that I am thinking of the skill*, 5 = *perfectly clear and vivid*).

### Data analysis

Data analysis was performed using R (version 4.4.0), RStudio (version 2024.04.2), and the lme4 package (Bates et al., 2015) for main analyses. Significance level was set to *p* <.05.

#### Outlier analysis

Outlier analysis for the 9,000 movement time observations for forward movements in the CRFT followed a top-down approach from participant to movement times (Aguinis et al., 2013), focusing on lower levels to detect errors such as double space bar presses. This resulted in removing *n* = 204 (2.23%) observations based on univariate analysis (boxplots, histograms, *SD* analysis with a 2.24 *SD* threshold based on M. A. Martin & Roberts, 2010). After inspecting the results of the multivariate Mahalanobis distance analysis, only *n* = 2 more outliers were removed, which were inconsistent with a participant’s behavior.

#### AIA score

For each tool condition, four AIA scores were calculated, representing the absolute values derived from (1) the absolute deviation approach (Eq. 1), (2) the relative deviation approach (Eq. 2), and (3) the constraint approach (Eq. 3). Using absolute values ensured that slower or faster action imagery than action execution was weighted equally. Before calculating the AIA scores based on (4) the Fitts’ law approach, movement times were log-transformed, correcting for its positive skew. A linear regression was computed for each participant’s data ($\mathrm{M}\mathrm{T}=a+b\times \mathrm{m}\mathrm{o}\mathrm{v}\mathrm{e}\mathrm{m}\mathrm{e}\mathrm{n}\mathrm{t}\text{ } \mathrm{d}\mathrm{i}\mathrm{f}\mathrm{f}\mathrm{i}\mathrm{c}\mathrm{u}\mathrm{l}\mathrm{t}\mathrm{y}$). Then, we calculated the AIA scores using Eq. 6, which represents the relative deviation of the standardized effects (β; derived from slopes *b*) of movement difficulty on movement time MT between action execution and action imagery within a tool condition:

6 $$\mathrm{A}\mathrm{I}\mathrm{A}\text{ }\mathrm{s}\mathrm{c}\mathrm{o}\mathrm{r}\mathrm{e}= \left|1-\frac{{\upbeta}_{\mathrm{A}\mathrm{E}}}{{\upbeta}_{\mathrm{A}\mathrm{I}}}\right|$$

A smaller deviation in the standardized effects β results in a lower AIA score, indicating a greater ability to imagine movements according to the manipulation of movement difficulty. Conceptually, an AIA score of 0 indicates that changes in movement difficulty affect duration of action execution and action imagery equally. The use of standardized regression coefficients (β) highlights relative deviations rather than absolute deviations. This ensures that individuals are not penalized for a systematic deviation from real-time action imagery (given the uncertainty of their meaningfulness), but rather for an inaccurate representation of movement difficulty in their action imagery.

#### Comparison of mental chronometry approaches

To evaluate the sensitivity of each mental chronometry approach to interindividual differences, we analyzed the distributions of AIA scores derived from the absolute deviation, relative deviation, constraint, and Fitts’ law approaches based on their skewness and kurtosis. We assessed the convergent validity of AIA scores across tools using Spearman correlations and correlated AIA scores and subjective measures. Spearman instead of Pearson correlations were used for non-normal AIA scores. Correlation coefficients were interpreted based on their magnitude (Cohen, 1988) as weak (≥.10), moderate (≥.30), strong (≥.50), or very strong (≥.70).

#### Statistical approach

A comparison of the intercept-only model with a random intercept model justified the multilevel approach (overall model, χ^2^(1) = 2,719.8, *p* <.001). Accounting for repeated measures, generalized linear mixed models (GLMMs) were fitted using maximum likelihood estimation with a Laplace approximation (level 1: movement time, level 2: participant). The GLMMs employed a gamma distribution with an identity link function, assuming that manipulations of movement difficulty and CRFT condition directly influence movement times. This approach allowed us to use raw movement times in milliseconds (Lo & Andrews, 2015), while effectively managing heteroskedasticity and non-normality of residuals. Movement difficulty was grand-mean centered, resulting in movement difficulty ranging from −2 (easiest movement) to +2 (hardest movement). Categorical predictors with two levels (action, tool) were effect-coded (−0.5, +0.5), so that coefficients represent average effects across participants, controlling for fixed and random effects. The GLMMs were built up sequentially. Following our expectations, fixed effects, interaction effects, and random slopes were added in a stepwise manner. In the model selection process, the Akaike information criterion (Akaike, 1974) was used to identify the optimal model.

## Results

### Testing the Fitts’ law approach across tools

**Fig. 2:**
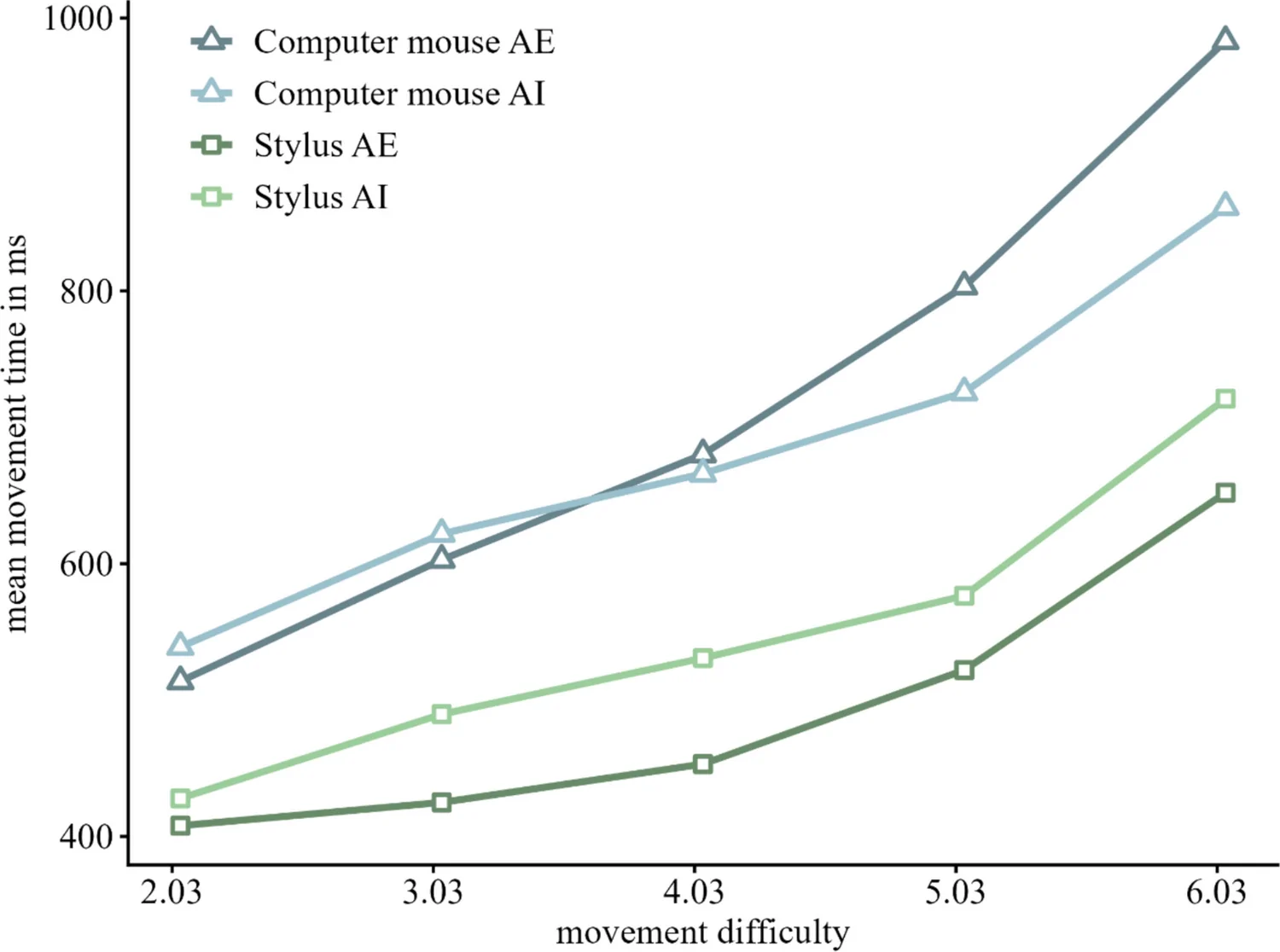
Mean movement time (in ms) per CRFT condition and movement difficulty. *AE* action execution; *AI* action imagery

The line graph in Fig. 2 displays the mean movement times across CRFT conditions and movement difficulties (for corresponding boxplots per movement difficulty and CRFT condition, see Fig. A in the Supplementary Material). Guided by our expectations, we sequentially fitted a GLMM incorporating all CRFT conditions (overall model), thereby comparing it to simpler models (see Table A in the Supplementary Material). The overall best-fitting model included fixed effects for movement difficulty (assuming a linear effect on movement time; slope *b* in Eq. 4), tool condition (assuming slower movement times for the computer mouse condition), action condition (assuming differences between execution durations and imagery durations), and random slopes for movement difficulty (expecting interindividual differences in the effect of movement difficulty on movement time). To reflect our expectation that changes in movement difficulty would have a larger impact on movement time in the computer mouse conditions, we added a movement difficulty × tool interaction. Expecting larger absolute differences in movement times between executed and imagined movements in the computer mouse condition, we included a tool × action interaction. In an exploratory manner, we incorporated a three-way movement difficulty × tool × action interaction to observe whether movement difficulty influenced movement times in action execution and action imagery differently across tools. This required adding a movement difficulty × action interaction that could reveal whether movement difficulty affected movement time more/less in one of the action conditions. Post hoc, we fitted four GLMMs, each comparing two specific CRFT conditions. These post hoc models tested the main effects of movement difficulty and CRFT condition (effect-coded as action and tool) and their interaction. Like the overall model, each post hoc model included a random slope for movement difficulty and a random intercept for participants. In a separate sensitivity analysis, we added action imagery experience as a covariate to the overall model, which did not alter the pattern of results (see Table B in the Supplement).

#### Tool-dependent differences in execution and imagery durations

Results of the final overall model can be obtained from Table 1. In line with expectations, movement times with the computer mouse were slower than with the stylus, as reflected by the significant main effect of tool. The main effect of tool was modified by a significant interaction with action. As expected, a paired-samples *t*-test indicated a significantly larger deviation in absolute movement times between the action conditions when using the computer mouse (*M* ± *SD* = 171 ± 113 ms) compared to the stylus (*M* ± *SD* = 95 ± 105 ms), *t*(29) = 3.5, *p* <.001, with a mean difference of 76 ms (95% CI [38; ∞]).

**Table 1:** Results of the final overall generalized linear mixed model on movement time (in ms) with movement difficulty, tool condition, action condition, and their interactions as independent variables

	Fixed effects					
Overall model	*b*	*SE*		*t*		*p*
Intercept	631	5		137.6		<.001
Movement difficulty	78	4		22.1		<.001
Tool	174	3		67.4		<.001
Action	28	2		11.8		<.001
Movement difficulty × tool	31	2		18.5		<.001
Tool × action	94	4		23.1		<.001
Movement difficulty × action	24	2		14.9		<.001
Movement difficulty × tool × action	45	3		13.5		<.001
	Random effects					
	*Var*		*SD*		*r*	
Participant (intercept)	1,840		43			
Movement difficulty (slope)	217		14		.60	

Participant as grouping variable

Movement difficulty = [−2; 2]

Tool: −0.5 = stylus, 0.5 = computer mouse

Action: −0.5 = action imagery, 0.5 = action execution

The significant main effect of action indicated faster action imagery than action execution when controlling for the included fixed and random effects, but movement time patterns diverged across tools: In line with expectations, movement times deviated significantly from real-time action imagery in both tool conditions. When using the stylus, movement times were significantly slower in action imagery than in action execution, as indicated by the significant main effect of action in the post hoc model (post hoc model 1 in Table 2; for random slopes see Table C in the Supplement). When using the computer mouse, movement times were significantly faster in action imagery than in action execution, as indicated by the significant main effect of action (post hoc model 2). However, action interacted significantly with movement difficulty, such that movement difficulty had a weaker effect on movement time in action imagery compared to action execution (i.e., shallower slope *b*). For easier movements with the computer mouse, action imagery was slower than action execution (aligning with the stylus conditions), but for more difficult movements, action imagery was faster than action execution (illustrated by the crossover point in Fig. 2 between movement difficulty = 3.03 and movement difficulty = 4.03).

**Table 2:** Post hoc generalized linear mixed models testing differences in movement time (in ms) and slopes *b* between CRFT conditions

Post hoc models	*b*	*SE*	*t*	*p*
1: Stylus AE vs. stylus AI				
Intercept	550	5	117.5	<.001
Movement difficulty	63	4	15.5	<.001
Action	–23	3	–9.2	<.001
Movement difficulty × action	–0	2	–0.2	.85
2: Computer mouse AE vs. computer mouse AI				
Intercept	734	10	72.4	<.001
Movement difficulty	98	4	24.4	<.001
Action	99	3	28.5	<.001
Movement difficulty × action	53	2	20.6	<.001
3: Computer mouse AE vs. stylus AE				
Intercept	622	6	95.9	<.001
Movement difficulty	83	4	21.9	<.001
Tool	230	3	82.2	<.001
Movement difficulty × tool	56	2	29.2	<.001
4: Computer mouse AI vs. stylus AI				
Intercept	657	7	97.5	<.001
Movement difficulty	76	5	16.7	<.001
Tool	107	3	33.3	<.001
Movement difficulty × tool	4	2	1.8	.08

Participant as grouping variable

Movement difficulty = [–2; 2]

Tool: –0.5 = stylus, 0.5 = computer mouse

Action: –0.5 = action imagery, 0.5 = action execution

While the slopes *b* of the respective action conditions differed when using the computer mouse (movement difficulty × action in post hoc model 2) but not the stylus (movement difficulty × action in post hoc model 1), further exploratory post hoc analyses revealed that the slopes *b* between the tools differed significantly in action execution (significant movement difficulty × tool interaction in post hoc model 3), but not in action imagery (no significant movement difficulty × tool interaction in post hoc model 4). This pattern of results remained unchanged after Bonferroni–Holm correction of the post hoc interaction tests involving movement difficulty (corrected *p*s <.001 for significant and >.15 for nonsignificant interactions). Figure 2 illustrates the results pattern, showing diverging lines for action execution and parallel lines for action imagery across tools. Hence, although we observed the expected significant movement difficulty × tool interaction in the overall model, this stronger effect of movement difficulty on movement time when using the computer mouse compared to the stylus was evident in action execution only (not in action imagery). This explains the observed significant three-way interaction, reflecting a particularly pronounced effect of movement difficulty on movement time for computer mouse AE.

#### Adherence to Fitts’ law across action conditions and tool conditions

Separate GLMMs were fitted for each CRFT condition to assess compliance with Fitts’ law. Each model incorporated a fixed effect for movement difficulty, a random intercept and a random slope for movement difficulty, grouped by participant. These models were then compared to simpler models that did not include a random slope for movement difficulty. In additional alternative models, movement difficulty was replaced with target width *W* (fixed effect and random slope). With the exception of the model for the stylus AI condition, all models were singular. Thus, simpler models without a random slope *W* were fitted for all CRFT conditions. Across all CRFT conditions, the initial model, which included a fixed effect and random slope for movement difficulty, outperformed alternatives (see Table D in the Supplement), thereby providing support for variability in slopes *b* across participants. The fixed effects of movement difficulty were significant in each CRFT condition (computer mouse AE, *b* = 111; computer mouse AI, *b* = 89; stylus AE, *b* = 57; stylus AI, *b* = 75; *p*s <.001), thereby providing support for the assumption that Fitts’ law holds in all CRFT conditions. However, slight deviations from linearity are illustrated in Fig. 2, particularly at movement difficulty = 6.03.

### Testing the Fitts’ law approach against alternative mental chronometry approaches

Because the slopes for movement difficulty across participants were significant and showed sufficient variability in all models, we could assume sufficient interindividual differences in slopes *b* and thus the AIA scores. Table 3 presents a comparison of AIA scores obtained from the absolute deviation, relative deviation, constraint, and Fitts’ law approaches to mental chronometry. The stylus AIA scores from the absolute, relative deviation, and constraint approach deviated from normality, as indicated by skewness > 2 and kurtosis > 7 (e.g., Curran et al., 1996; Ryu, 2011). The other AIA scores approximated the normal distribution. In all but the relative deviation approach, participants performed significantly worse in the computer mouse condition than the stylus condition, as indicated by larger AIA scores. Notably, only the Fitts’ law approach yielded significant convergent validity in AIA scores across tools (see Spearman correlations).

**Table 3:** Distribution and convergent validity of absolute, relative, constraint, and Fitts’ law action imagery ability scores across tool conditions

	Absolute deviation		Relative deviation		Constraint		Fitts’ law	
	Stylus	Computer mouse	Stylus	Computer mouse	Stylus	Computer mouse	Stylus	Computer mouse
*M*	95	171	0.20	0.24	0.26	0.48	0.27	0.69
*SD*	105	113	0.22	0.16	0.28	0.24	0.28	0.86
Skewness	2.60	0.62	2.51	0.42	2.24	0.03	1.58	1.77
Kurtosis	10.55	2.97	9.75	2.38	7.92	2.49	5.61	5.20
*t*-test, *p*	<.001		.13		<.001		<.01	
Spearman correlation								
ρ	−.01		.05		.05		.51	
*p*	.96		.81		.80		<.01	

*t*-tests between tool conditions were one-sided

### Exploratory correlations between mental chronometry approaches and subjective action imagery quality

In additional exploratory analyses, we examined whether, consistent with previous research on the relation between subjective measures and behavioral tasks (e.g., Dahm et al., 2022; Moreno-Verdú et al., 2026; Muraki & Pexman, 2021; Suggate, 2024; Williams et al., 2015), a pattern of discriminant validity would emerge between subjective action imagery quality and behaviorally measured action imagery ability in the CRFT. This furthermore allowed us to explore whether this relationship depended on the tool and mental chronometry approach used. For AIA scores that approximated normality, we calculated Pearson correlations (|*r*_crit_|=.36) with action imagery experience, the VMIQ-2 and its subscales (external visual, internal visual, kinesthetic action imagery), and the congruent action imagery items. For AIA scores that deviated from normality, we used Spearman correlations (|ρ_crit_|=.37). Since lower AIA scores across all approaches reflected higher action imagery ability, negative correlations with the VMIQ-2, its subscales and the action imagery items would indicate convergent validity with subjective action imagery ability. However, we observed significant *positive* correlations between the VMIQ-2 (total score and subscales) and AIA scores derived from the absolute and relative deviation approaches in the stylus condition (see Table 4).

**Table 4:** Correlations of action imagery experience and the VMIQ-2 and its subscales with absolute deviation, relative deviation, constraint, and Fitts’ law action imagery ability scores

	Absolute deviation		Relative deviation		Constraint		Fitts’ law	
Variables	Stylus^a^	Computer mouse^b^	Stylus^a^	Computer mouse^b^	Stylus^a^	Computer mouse^b^	Stylus^b^	Computer mouse^b^
Experience	.14	–.27	.16	–.30	.10	–.08	–.06	.03
VMIQ-2	**.59**	.06	**.60**	.07	–.06	.22	–.02	.09
External visual	**.39**	.10	**.38**	.09	–.08	.34	–.15	.27
Internal visual	**.50**	.08	**.51**	.09	–.11	.12	.09	–.17
Kinesthetic	**.55**	–.02	**.56**	–.01	–.10	.09	.00	.13

Significant values shown in bold

^a^ Spearman correlation. ^b^ Pearson correlation

Additionally, in the stylus condition, relative deviation AIA scores correlated significantly and positively with the kinesthetic action imagery item (ρ =.39, *p* =.04). In the computer mouse condition, a significant positive correlation was found between the constraint AIA scores and the visual action imagery item (*r* =.51, *p* <.01). A significant *negative* correlation resulted for Fitts’ law AIA scores in the stylus condition and the ease of generating the action imagery (see Table 5). All correlations between AIA scores derived from the absolute deviation, relative deviation, and constraint approaches (Table E) and with the action imagery items (Table F) can be obtained from the Supplementary Material.

**Table 5:** Mean and standard deviation of action imagery items and their Pearson correlation with Fitts’ law action imagery ability scores in the congruent tool condition

	Stylus			Computer mouse		
Item	*M*	*SD*	*r*	*M*	*SD*	*r*
Ease	7.00	2.16	**–.48**	6.47	1.83	.05
Speed	6.13	2.53	–.22	6.10	2.54	–.07
Maintenance	6.80	1.95	–.13	6.60	2.16	–.26
Control	7.40	3.08	–.12	7.00	2.03	–.09
Clarity	6.93	1.95	–.15	6.80	2.12	–.29
Visual	6.47	2.50	–.27	6.77	2.43	.05
Kinesthetic	5.57	1.98	–.10	5.40	2.34	–.27
Tactile	7.40	2.34	.00	7.53	1.72	–.07
Auditory	3.80	1.25	.13	3.83	2.90	.13

Significant values shown in bold

## Discussion

In the present study, we demonstrated the use of a Fitts’ law-based mental chronometry approach to measure action imagery ability related to imagery manipulation and timing. In the CRFT, movement difficulty was manipulated within randomized blocks of action execution and action imagery. We examined how movement difficulty was reflected in execution versus imagery durations, forming the basis of the Fitts’ law AIA score. We evaluated whether this Fitts’ law approach provides converging results across tools differing in familiarity and complexity: a stylus and a computer mouse. All four CRFT conditions (stylus AE, stylus AI, computer mouse AE, and computer mouse AI) adhered to Fitts’ law. Overall, movement times were longer in the computer mouse conditions than in the stylus conditions. However, while in the stylus conditions imagery durations exceeded execution durations, movement difficulty interacted with action condition in the computer mouse conditions. Shallower slopes resulted for computer mouse AI compared to computer mouse AE, such that at higher movement difficulties, imagery durations became shorter than execution durations. Although slopes differed across action execution conditions, slopes did not differ across action imagery conditions. When comparing the derived AIA scores, only the Fitts’ law approach provided convergent validity for AIA scores across tools, thereby outperforming absolute deviation, relative deviation, and constraint approaches.

### Testing the Fitts’ law approach against alternative mental chronometry approaches

By comparing GLMMs with different explanations of the relationship between movement difficulty and movement time (e.g., using target width instead of movement difficulty as independent variable), we demonstrated that Fitts’ law is applicable to executed and imagined movements with different tools. This pattern suggests that action imagery preserves functional relations of action execution (Fitts, 1954). Action imagery hence likely involves processes that also contribute to action execution (Jeannerod, 2001; Rieger et al., 2024), rather than relying solely on knowledge or an estimation of movement times. Adherence to Fitts’ law during action imagery is unlikely to be explained by reliance on eye movements rather than mental action simulation. Although eye movements support action imagery of aiming movements (Pathak et al., 2023), movement difficulty was manipulated through target width, whereas the distance covered by eye movements remained constant. Primary reliance on eye movements would therefore not be expected to produce imagery durations that varied systematically with movement difficulty as specified in Fitts’ law. Sufficient interindividual differences in slopes (random slopes for the effect of movement difficulty on movement time) and Fitts’ law AIA scores further indicated that the approach can differentiate individuals’ action imagery ability. The CRFT therefore appears well suited to assessing action imagery ability components using the Fitts’ law AIA score.

We tested the Fitts’ law approach across tool conditions within the CRFT that varied in familiarity and complexity and, in turn, in task demands. As expected, the computer mouse condition was more demanding than the stylus condition, which was reflected in longer execution and imagery durations, larger absolute deviations between execution and imagery durations, and higher AIA scores. This provided a basis for testing which mental chronometry approaches would show convergent AIA scores across tool conditions, given that both should reflect individuals’ action imagery ability. When using the stylus, AIA scores derived from the absolute deviation, relative deviation, and constraint approaches were positively skewed, indicating limited sensitivity to differentiate high-performing participants. This pattern was absent when using the more demanding computer mouse and for AIA scores derived from the Fitts’ law approach across tools. Notably, the Fitts’ law approach showed convergent validity of AIA scores across tools, while the alternative approaches did not. These findings suggest that the Fitts’ law approach, compared to alternative mental chronometry approaches, is more sensitive to individual differences in action imagery ability and better captures variance that is shared across tool conditions.

In addition to the lack of convergence across tools, the exploratory associations between AIA scores and subjective imagery vividness suggest that the absolute deviation and relative deviation approaches are sensitive to influences on imagery duration that are not specific to action imagery ability. Earlier research found no association between absolute deviation-based mental chronometry and self-reported ease of imagining movements (Williams et al., 2015). The present findings go further: higher subjective vividness on the VMIQ-2 was associated with poorer AIA scores derived from the absolute and relative deviation approaches (stylus conditions). Bias in self-evaluation may partly explain these results. Individuals with lower actual ability tend to overestimate their performance, whereas individuals with higher ability may underestimate their performance when they compare themselves with others and assume that they performed similarly well (Kruger & Dunning, 1999; Ross et al., 1977). However, this cannot fully explain the present findings, because comparable positive associations were not evident for the Fitts’ law AIA scores. One way in which deviation-based approaches may capture influences beyond action imagery ability is through variation in how participants balance speed and accuracy. Such variation is likely under instructions to respond “as fast and accurately as possible” (Heitz, 2014). Higher subjective vividness may be associated with a stronger emphasis on accuracy and, consequently, slower action imagery. This interpretation is consistent with evidence that vividly seeing and feeling one’s hands in behavioral tasks can impair performance when speed and accuracy must be combined (Dahm & Sachse, 2025). It also fits the present results, in which participants on average rated their imagery as clear and reasonably vivid (about 4 out of 5), yet action imagery was slower than action execution in the stylus conditions. Although slower imagery may reflect more detailed and accuracy-oriented simulation (Calmels & Fournier, 2001; Reed, 2002), deviation-based approaches treat such slowing as lower action imagery ability, even when imagery more accurately preserves the relation between movement difficulty and movement time. The Fitts’ law approach reduces this bias by focusing on the extent to which action imagery preserves the relation between movement difficulty and movement time, rather than on deviation from real-time action imagery. This may explain why Fitts’ law AIA scores showed a less contradictory pattern of associations with subjective measures. Although they did not correlate with the more general vividness ratings of the VMIQ-2, tendencies toward negative associations with task-specific ratings of action imagery quality were observed.

### Theoretical implications of tool-related differences in execution and imagery durations

Although the movements were closed and repetitive (Guillot & Collet, 2005; Guillot et al., 2012), real-time action imagery was not achieved in stylus AI. Imagery durations exceeding execution durations with the stylus aligns with prior findings on the mental chronometry of short movements (Dahm & Rieger, 2016b; Debarnot et al., 2012; Grealy & Shearer, 2008; Orliaguet & Coello, 1998), and findings in pointing tasks involving horizontal movements only (Cerritelli et al., 2000; Maruff et al., 1999b; Wilson et al., 2001). However, it contrasts with findings of execution durations exceeding imagery durations in spatially more complex pointing tasks involving radial movements (Caeyenberghs et al., 2009a, 2009b; Ferguson et al., 2015; Smits-Engelsman & Wilson, 2013). This discrepancy may be due to methodological differences. Because the difficulty of return movements was constant, the CRFT isolated movement times of forward movements, whereas movement times in earlier tasks reflected the duration of the whole movement sequence. In addition, participants lifted the stylus after each tap, which introduced a three-dimensional component and likely increased the complexity of the movements to be imagined. The opposite direction of average deviations from real-time action imagery in earlier radial pointing tasks and the CRFT therefore suggests that deriving action imagery ability from such deviations requires careful consideration of methodological and task-specific influences.

Following Glover and Baran’s (2017) motor-cognitive model, stylus AI taking longer than stylus AE may result from the costs associated with switching attention between action imagery and the response (here: space bar presses) used to index it, which becomes more pronounced with shorter movements. At the same time, the motor-cognitive model cannot explain the observation that, specifically for higher movement difficulties, computer mouse AI was shorter than computer mouse AE. If we assume that computer mouse AI holds lower fidelity than stylus AI, subtracting the execution duration from the imagery duration should result in a larger *positive* (instead of negative) difference in the computer mouse condition than in the stylus condition. Spinning this further, the motor-cognitive model would predict that, across tool conditions, movements requiring higher precision (here: greater movement difficulty) should involve greater conscious elaboration, resulting in disproportionately longer durations (i.e., steeper slope) in action imagery than action execution. We did not observe this pattern, even in the absence of complex tool demands such as those posed by the computer mouse. There was no interaction between movement difficulty and action condition in the stylus conditions. This might be explained by the level of task automation in the concurrent task (space bar presses). Tapping with the stylus and simultaneously pressing the spacebar might have been automatic enough to avoid further depletion of executive resources. While the motor-cognitive model might apply well to tools with only minor unfamiliar properties, its applicability to more complex tool use may be limited.

Rather than aligning with predictions from the motor-cognitive model, our results for the computer mouse conditions are more consistent with findings by Macuga et al. (2012). They showed that both action execution and action imagery adhered to Fitts’ law, even when using novel tools (e.g., regular, top-heavy, or bottom-heavy pens). In their study, participants’ imagery durations adapted to the novel tools, although imagery durations were shorter than execution durations. This led to a smaller overall slope for imagined versus executed movements; as observed in the computer mouse conditions of the present study.

In our study, while slopes did not differ between stylus AE and stylus AI or between stylus AI and computer mouse AI, they did differ between stylus AE and computer mouse AE and between computer mouse AE and computer mouse AI. This pattern suggests that although participants were aware of the increased difficulty associated with using a computer mouse (reflected in longer durations), they struggled to update their internal model of the movement to fully integrate the computer mouse’s unique properties (e.g., speed, acceleration, sensitivity)—properties that likely contributed to significantly steeper slopes in computer mouse AE (e.g., through oversteering, need for higher precision).

That is, despite a familiarization phase, the internal model was not sufficiently adapted to account for the computer mouse’s properties. Successfully hitting targets with a novel or complex tool may rely strongly on sensory feedback from actual effects (Blakemore et al., 2002; Rieger et al., 2024). Compared to action execution, such feedback is absent during action imagery, making tool-specific errors like oversteering less likely to be detected and corrected (Dahm & Rieger, 2019a, 2019b). This became particularly pronounced under conditions of higher precision (larger movement difficulties), resulting in greater discrepancies between execution and imagery durations. It appears that, for complex tools like the computer mouse, predicted effects alone are insufficient to compensate for the lack of sensory input during action imagery (for a discussion, see Rieger et al., 2024). This subsequently explains why (1) the computer mouse AIA scores were significantly worse (i.e., larger) than the stylus AIA scores, and (2) the effect of movement difficulty on movement time did not differ between stylus AI and computer mouse AI. It is therefore likely that, apart from explicit knowledge of increased difficulty of hitting the targets (longer durations), participants mainly relied on the internal model of executing the movement without the computer mouse, thus “mimicking” stylus AI. That is, they likely relied on the internal model of a more familiar action (Rieger, 2012), converging with research indicating that experience with the specific movement to be imagined influences the accuracy of imagery durations (Yoxon et al., 2015).

Taken together with the strong but insufficient convergent validity (to assume the measurement of the exact same construct) between AIA scores across tools, and the missing convergent validity of AIA scores and subjective action imagery ratings within the computer mouse conditions, these results indicate that we may not have measured action imagery ability per se in the computer mouse conditions. Instead, we likely captured the ability to integrate the computer mouse’s properties into the internal model of the movement—which we assume is dependent on action imagery ability, thereby explaining the convergent validity of AIA scores.

A contributing factor to the difficulty in executing and imagining clicking the targets may have been the default mouse cursor acceleration. When strongly accelerating the computer mouse, the amplitude of the physically executed movement of the hand and the amplitude of the movement of the cursor on the screen can decouple. Additionally, while participants could see their hand movements during stylus AE, these movements were outside their view in computer mouse AE, making visual action imagery of their own hand more difficult, as they visually focused on the targets on the screen. Consequently, visual and kinesthetic sensations may contradict when using the computer mouse in action imagery, thereby impeding the integration of multiple sensory components in action imagery (Krüger et al., 2022). Rather than imagining the physical movement performed (i.e., moving the computer mouse on the table), participants may have primarily imagined the action effects (i.e., the movements of the cursor on the screen; Dahm & Sachse, 2025).

Beyond tool properties, the need to translate horizontal movements on the table into cursor movements on a vertical screen may have contributed to greater difficulty in the computer mouse condition. However, forward movements corresponded to upward movements on the screen, maintaining visual field compatibility. Given visual field compatibility, control display compatibility (control movements in the same and parallel direction as cursor movements) was shown not to provide an additional benefit for visuomotor performance (Worringham & Beringer, 1998). The student sample’s probable familiarity with the required mapping further suggests that tool properties influenced movement times in computer mouse AE more strongly than the required mapping. Still, performance decrements in such mappings can be particularly pronounced when visual feedback is limited (Ghilardi et al., 1995). This aligns with the lower accuracy of computer mouse AI compared to stylus AI and supports the interpretation that predicted effects alone are insufficient to compensate for the lack of actual feedback (Rieger et al., 2024).

### Strengths and limitations

The present findings are strengthened by methodological refinements of the CRFT that reduce limitations of earlier radial pointing paradigms (e.g., Caeyenberghs et al., 2009a; Smits-Engelsman & Wilson, 2013). Specifically, we used fully randomized blocks of action execution and action imagery trials rather than alternating execution and imagery within each level of movement difficulty, thereby reducing memory reliance during action imagery. In previous studies, the start and end of executed and imagined movements were indicated through movements with the dominant hand toward designated visual targets. The CRFT avoids this immediate action execution before action imagery with the same hand by requiring spacebar presses with the nondominant hand for each executed and imagined tap or click. This made it possible to isolate the duration of forward movements rather than measuring the duration of the whole movement sequence, given that return movements did not vary in difficulty, while increasing the number of observations.

While our findings provide support for the Fitts’ law approach to mental chronometry, some limitations should be considered. First, the absence of significant correlations between stylus AIA scores derived from our Fitts’ law approach and the VMIQ-2 may be viewed as a limitation. However, this result aligns with prior research suggesting that subjective and behavioral measures (mental chronometry: Moreno-Verdú et al., 2026; Schott, 2013; Williams et al., 2015; mental rotation: Dahm et al., 2022; imagery-stimulus comparison: Muraki & Pexman, 2021; Schott, 2013; mental comparison: Suggate, 2024) capture different components of action imagery ability. Although our sample size did not allow us to detect small effects, the focus of our convergent validity analysis was not on detecting weak correlations. Moreover, the direction of correlations for the absolute deviation, relative deviation, and constraint approaches indicated discriminant validity.

Second, the validity of the Fitts’ law AIA score depends on a linear relationship between movement difficulty and movement time. This assumption may not hold at extreme levels of movement difficulty, as suggested by the slight graphical deviation from linearity at the highest movement difficulty in the present study and by earlier research (e.g., Buck, 1986; Crossman & Goodeve, 1983; Knight & Dagnall, 1967). Extreme movement difficulties should therefore be avoided in future applications. While our approach is designed for healthy populations in which adherence to Fitts’ law can be expected, previous findings indicate that action execution of participants with developmental coordination disorder preserves Fitts’ law (Maruff et al., 1999b; Wilson et al., 2001). A lack of linearity in action imagery itself may therefore indicate impaired action imagery ability.

Third, the Fitts’ law AIA score involves several levels of abstraction, making it less intuitive than deviation-based approaches. However, it is intended to reduce potential confounds by drawing on theoretical and empirical work on lawful relations between action execution and action imagery (Fitts, 1954) and by accounting for evidence that imagery durations are systematically influenced by factors not necessarily related to action imagery ability (e.g., Reed, 2002). Moreover, our results show that apparent interpretability, as in deviation-based approaches, can be misleading with respect to validity.

### The CRFT as a measure of action imagery ability

Beyond highlighting the limitations of using deviations from real-time action imagery as a measure of individuals’ action imagery ability and evaluating the Fitts’ law approach as an alternative, our study points to the value of standardizing behavioral tasks such as the CRFT. Standardization of behavioral tasks across studies supports the development of normative cutoff values for impaired action imagery ability, ensuring meaningful interpretation of scores and supporting the indication and tailoring of action imagery practice. The CRFT provides an objective measure of action imagery ability related to action imagery manipulation and timing, grounded in research on functional similarities between action execution and action imagery (approximated via Fitts’ law, 1954). A ready-to-use version of the task is freely available on GitHub with adjustable settings for specific applications (e.g., language): https://github.com/carlacz/CRFT. Apart from a standard computer and keyboard, the CRFT requires a fine-tipped stylus and a screen providing a stable tapping surface (we hence recommend using a touchscreen monitor). Given this experimental setup, the CRFT is applicable to laboratory and applied settings. Future research could advance understanding of action imagery by examining the CRFT’s convergent validity with other behavioral tasks that assess distinct components of action imagery ability (e.g., Madan & Singhal, 2013; Moreno-Verdú et al., 2025; Suggate, 2024), thereby clarifying its multidimensional structure.

## Conclusion

In this paper, we critically examined the assumption of real-time action imagery in mental chronometry tasks and demonstrated that this assumption can undermine the validity of resulting AIA scores. We proposed a Fitts’ law–based approach that addresses associated limitations and outperformed established and earlier proposed alternatives in a study with the CRFT: the absolute deviation, relative deviation, and constraint approaches. Our findings support the view that action imagery involves processes from action execution while illustrating the complex interplay of factors influencing imagery durations. Specifically, whether imagery durations exceed execution durations in short movements depends on the tool used. Action imagery with a complex tool (e.g., a computer mouse) appeared to draw on internal models developed for more familiar tools (e.g., a stylus). This suggests that mental chronometry tasks using complex tools may fall prey to reflecting an individual’s ability to integrate the tool’s properties into the internal model of the movement rather than action imagery ability per se. Therefore, selecting sufficiently familiar movements and tools is critical to ensure that mental chronometry tasks validly measure action imagery ability. Our results further support the notion that mental chronometry tasks capture distinct components of action imagery ability—components that are not reflected in commonly used subjective measures.

## Supplementary Information

Below is the link to the electronic supplementary material.

Supplementary file1 (PDF 291 kb)

## Declarations

### Open practices statement

The data, experiment file, and code for data analyses are published on the Open Science Framework: https://osf.io/t7xjc/

The experiment was not preregistered.

## References

[CR1] Aguinis, H., Gottfredson, R. K., & Joo, H. (2013). Best-practice recommendations for defining, identifying, and handling outliers. *Organizational Research Methods,**16*(2), 270–301. 10.1177/1094428112470848

[CR2] Akaike, H. (1974). A new look at the statistical model identification. *IEEE Transactions on Automatic Control,**19*(6), 716–723. 10.1109/TAC.1974.1100705

[CR3] Alicke, M. D., Klotz, M. L., Breitenbecher, D. L., Yurak, T. J., & Vredenburg, D. S. (1995). Personal contact, individuation, and the better-than-average effect. *Journal of Personality and Social Psychology,**68*(5), 804–825. 10.1037/0022-3514.68.5.804

[CR4] Alicke, M. D., & Sedikides, C. (2009). Self-enhancement and self-protection: What they are and what they do. *European Review of Social Psychology,**20*(1), 1–48. 10.1080/10463280802613866

[CR5] Annett, J. (1996). On knowing how to do things: A theory of motor imagery. *Cognitive Brain Research,**3*(2), 65–69. 10.1016/0926-6410(95)00030-5 8713546

[CR6] Bach, P., Frank, C., & Kunde, W. (2022). Why motor imagery is not really motoric: Towards a re-conceptualization in terms of effect-based action control. *Psychological Research,**88*, 1790–1804. 10.1007/s00426-022-01773-w 36515699 PMC11315751

[CR7] Bai, Y., Brillinger, M., Karlinsky, A., Poliakoff, E., Welsh, T. N., & Gowen, E. (2025). Speed-accuracy trade-offs in action perception, motor imagery, and execution of hand movements in autistic and non-autistic adults. *Scientific Reports,**15*(1), Article 13255. 10.1038/s41598-025-97036-w 40247000 PMC12006536

[CR8] Bakker, M., de Lange, F. P., Stevens, J. A., Toni, I., & Bloem, B. R. (2007). Motor imagery of gait: A quantitative approach. *Experimental Brain Research,**179*(3), 497–504. 10.1007/s00221-006-0807-x 17211663

[CR9] Bates, D., Mächler, M., Bolker, B., & Walker, S. (2015). Fitting linear mixed-effects models using lme4. *Journal of Statistical Software,**67*(1), 1–48. 10.18637/jss.v067.i01

[CR10] Beauchet, O., Annweiler, C., Assal, F., Bridenbaugh, S., Herrmann, F. R., Kressig, R. W., & Allali, G. (2010). Imagined timed up & go test: A new tool to assess higher-level gait and balance disorders in older adults? *Journal of the Neurological Sciences,**294*(1), 102–106. 10.1016/j.jns.2010.03.021 20444477

[CR11] Blakemore, S.-J., Wolpert, D. M., & Frith, C. D. (2002). Abnormalities in the awareness of action. *Trends in Cognitive Sciences,**6*(6), 237–242. 10.1016/S1364-6613(02)01907-1 12039604

[CR12] Buck, L. (1986). Target location effects in tapping tasks. *Acta Psychologica,**62*(1), 1–13. 10.1016/0001-6918(86)90002-8

[CR13] Caeyenberghs, K., Tsoupas, J., Wilson, P. H., & Smits-Engelsman, B. C. M. (2009a). Motor imagery development in primary school children. *Developmental Neuropsychology,**34*(1), 103–121. 10.1080/87565640802499183 19142769

[CR14] Caeyenberghs, K., Wilson, P. H., Van Roon, D., Swinnen, S. P., & Smits-Engelsman, B. C. M. (2009b). Increasing convergence between imagined and executed movement across development: Evidence for the emergence of movement representations. *Developmental Science,**12*(3), 474–483. 10.1111/j.1467-7687.2008.00803.x 19371372

[CR15] Calmels, C., & Fournier, J. (2001). Duration of physical and mental execution of gymnastic routines. *Sport Psychologist,**15*(2), 142–150. 10.1123/tsp.15.2.142

[CR16] Calmels, C., Holmes, P., Lopez, E., & Naman, V. (2006). Chronometric comparison of actual and imaged complex movement patterns. *Journal of Motor Behavior,**38*(5), 339–348. 10.3200/JMBR.38.5.339-348 16968679

[CR17] Carlson, K. D., & Herdman, A. O. (2012). Understanding the impact of convergent validity on research results. *Organizational Research Methods,**15*(1), 17–32. 10.1177/1094428110392383

[CR18] Cerritelli, B., Maruff, P., Wilson, P., & Currie, J. (2000). The effect of an external load on the force and timing components of mentally represented actions. *Behavioural Brain Research,**108*(1), 91–96. 10.1016/S0166-4328(99)00138-2 10680761

[CR19] Choudhury, S., Charman, T., Bird, V., & Blakemore, S.-J. (2007). Adolescent development of motor imagery in a visually guided pointing task. *Consciousness and Cognition,**16*(4), 886–896. 10.1016/j.concog.2006.11.001 17196830

[CR20] Cohen, J. (1988). *Statistical power analysis for the behavioral sciences* (2nd ed.). Lawrence Erlbaum Associates.

[CR21] Crossman, E. R. F. W., & Goodeve, P. J. (1983). Feedback control of hand-movement and Fitts’ law. *The Quarterly Journal of Experimental Psychology Section A,**35*(2), 251–278. 10.1080/14640748308402133 6571310

[CR22] Cumming, J., & Williams, S. E. (2012). The role of imagery in performance. *The Oxford Handbook of Sport and Performance Psychology* (pp. 213–232). Oxford University Press. 10.1093/oxfordhb/9780199731763.013.0011

[CR23] Curran, P. J., West, S. G., & Finch, J. F. (1996). The robustness of test statistics to nonnormality and specification error in confirmatory factor analysis. *Psychological Methods,**1*(1), 16–29. 10.1037/1082-989X.1.1.16

[CR24] Dahm, S. F. (2020). On the assessment of motor imagery ability: A research commentary. *Imagination, Cognition and Personality,**39*(4), 397–408. 10.1177/0276236619836091

[CR25] Dahm, S. F., Bart, V. K. E., Pithan, J. M., & Rieger, M. (2019). Deutsche Übersetzung und Validierung des VMIQ-2 zur Erfassung der Lebhaftigkeit von Handlungsvorstellungen [German translation and validation of the VMIQ-2 for the assessment of vividness of movement imagery]. *Zeitschrift Für Sportpsychologie,**26*(4), 151–158. 10.1026/1612-5010/a000273 32273834 PMC7145442

[CR26] Dahm, S. F., Muraki, E. J., & Pexman, P. M. (2022). Hand and foot selection in mental body rotations involves motor-cognitive interactions. *Brain Sciences,**12*, Article 1500. 10.3390/brainsci12111500 36358425 PMC9688262

[CR27] Dahm, S. F., & Rieger, M. (2016a). Cognitive constraints on motor imagery. *Psychological Research,**80*(2), 235–247. 10.1007/s00426-015-0656-y 25758054 PMC4629411

[CR28] Dahm, S. F., & Rieger, M. (2016b). Is there symmetry in motor imagery? Exploring different versions of the mental chronometry paradigm. *Attention, Perception & Psychophysics,**78*(6), 1794–1805. 10.3758/s13414-016-1112-9 PMC4972863 27173486

[CR29] Dahm, S. F., & Rieger, M. (2019a). Errors in imagined and executed typing. *Vision,**3*(4), Article 66. 10.3390/vision3040066 31756895 PMC6969896

[CR30] Dahm, S. F., & Rieger, M. (2019b). Is imagery better than reality? Performance in imagined dart throwing. *Human Movement Science,**66*, 38–52. 10.1016/j.humov.2019.03.005 30913415 PMC6520223

[CR31] Dahm, S. F., & Rieger, M. (2022). A little doubt saves many mistakes: Early and late error detection in copy-typing. *Open Psychology,**4*(1), 115–131. 10.1515/psych-2022-0006

[CR32] Dahm, S. F., & Sachse, P. (2025). Let’s do it: Response times in mental paper folding and its execution. *Quarterly Journal of Experimental Psychology,**78*(4), 731–743. 10.1177/17470218241249727 PMC11905326 38616184

[CR33] Davidson, P. R., & Wolpert, D. M. (2005). Widespread access to predictive models in the motor system: A short review. *Journal of Neural Engineering,**2*(3), 313–319. 10.1088/1741-2560/2/3/S11 16135891

[CR34] Debarnot, U., Sahraoui, D., Champely, S., Collet, C., & Guillot, A. (2012). Selective influence of circadian modulation and task characteristics on motor imagery time. *Research Quarterly for Exercise and Sport,**83*(3), 442–450. 10.1080/02701367.2012.10599879 22978194

[CR35] Decety, J., & Jeannerod, M. (1995). Mentally simulated movements in virtual reality: Does Fitts’s law hold in motor imagery? *Behavioural Brain Research,**72*(1), 127–134. 10.1016/0166-4328(96)00141-6 8788865

[CR36] Decety, J., Jeannerod, M., & Prablanc, C. (1989). The timing of mentally represented actions. *Behavioural Brain Research,**34*(1–2), 35–42. 10.1016/s0166-4328(89)80088-9 2765170

[CR37] Di Rienzo, F., Collet, C., Hoyek, N., & Guillot, A. (2012). Selective effect of physical fatigue on motor imagery accuracy. *PLoS ONE,**7*(10), Article e47207. 10.1371/journal.pone.0047207 23082148 PMC3474822

[CR38] Drury, C. G., & Woolley, S. M. (1995). Visually-controlled leg movements embedded in a walking task. *Ergonomics,**38*(4), 714–722. 10.1080/00140139508925143 7729399

[CR39] Ferguson, G. D., Wilson, P. H., & Smits-Engelsman, B. C. M. (2015). The influence of task paradigm on motor imagery ability in children with developmental coordination disorder. *Human Movement Science,**44*, 81–90. 10.1016/j.humov.2015.08.016 26319360

[CR40] Féry, Y.-A. (2003). Differentiating visual and kinesthetic imagery in mental practice. *Canadian Journal of Experimental Psychology / Revue Canadienne De Psychologie Expérimentale,**57*(1), 1–10. 10.1037/h0087408 12674365

[CR41] Fitts, P. M. (1954). The information capacity of the human motor system in controlling the amplitude of movement. *Journal of Experimental Psychology,**47*(6), 381–391. 10.1037/h0055392 13174710

[CR42] Fitts, P. M., & Peterson, J. R. (1964). Information capacity of discrete motor responses. *Journal of Experimental Psychology,**67*(2), 103–112. 10.1037/h0045689 14114905

[CR43] Ghilardi, M. F., Gordon, J., & Ghez, C. (1995). Learning a visuomotor transformation in a local area of work space produces directional biases in other areas. *Journal of Neurophysiology,**73*(6), 2535–2539. 10.1152/jn.1995.73.6.2535 7666158

[CR44] Glover, S., & Baran, M. (2017). The motor-cognitive model of motor imagery: Evidence from timing errors in simulated reaching and grasping. *Journal of Experimental Psychology: Human Perception and Performance,**43*(7), 1359–1375. 10.1037/xhp0000389 28368162

[CR45] Grealy, M. A., & Shearer, G. F. (2008). Timing processes in motor imagery. *European Journal of Cognitive Psychology,**20*(5), 867–892. 10.1080/09541440701618782

[CR46] Guillot, A., & Collet, C. (2005). Duration of mentally simulated movement: A review. *Journal of Motor Behavior,**37*(1), 10–20. 10.3200/JMBR.37.1.10-20 15642689

[CR47] Guillot, A., Hoyek, N., Louis, M., & Collet, C. (2012). Understanding the timing of motor imagery: Recent findings and future directions. *International Review of Sport and Exercise Psychology,**5*(1), 3–22. 10.1080/1750984X.2011.623787

[CR48] Hardwick, R. M., Caspers, S., Eickhoff, S. B., & Swinnen, S. P. (2018). Neural correlates of action: Comparing meta-analyses of imagery, observation, and execution. *Neuroscience and Biobehavioral Reviews,**94*, 31–44. 10.1016/j.neubiorev.2018.08.003 30098990

[CR49] Hétu, S., Grégoire, M., Saimpont, A., Coll, M.-P., Eugène, F., Michon, P.-E., & Jackson, P. L. (2013). The neural network of motor imagery: An ALE meta-analysis. *Neuroscience and Biobehavioral Reviews,**37*(5), 930–949. 10.1016/j.neubiorev.2013.03.017 23583615

[CR50] Heitz, R. P. (2014). The speed-accuracy tradeoff: History, physiology, methodology, and behavior. *Frontiers in Neuroscience,**8*, Article 150. 10.3389/fnins.2014.00150 24966810 PMC4052662

[CR51] Holmes, P., & Collins, D. J. (2001). The PETTLEP approach to motor imagery: A functional equivalence model for sport psychologists. *Journal of Applied Sport Psychology,**13*(1), 60–83. 10.1080/10413200109339004

[CR52] Huys, R., Knol, H., Sleimen-Malkoun, R., Temprado, J.-J., & Jirsa, V. K. (2015). Does changing Fitts’ index of difficulty evoke transitions in movement dynamics? *EPJ Nonlinear Biomedical Physics,**3*(1), 1–15. 10.1140/epjnbp/s40366-015-0022-4

[CR53] Jacobs, S., Danielmeier, C., & Frey, S. H. (2010). Human anterior intraparietal and ventral premotor cortices support representations of grasping with the hand or a novel tool. *Journal of Cognitive Neuroscience,**22*(11), 2594–2608. 10.1162/jocn.2009.21372 19925200

[CR54] Jeannerod, M. (1994). The representing brain: Neural correlates of motor intention and imagery. *Behavioral and Brain Sciences,**17*(2), 187–202. 10.1017/S0140525X00034026

[CR55] Jeannerod, M. (2001). Neural simulation of action: A unifying mechanism for motor cognition. *NeuroImage,**14*(1), 103–109. 10.1006/nimg.2001.0832 11373140

[CR56] Kilteni, K., Andersson, B. J., Houborg, C., & Ehrsson, H. H. (2018). Motor imagery involves predicting the sensory consequences of the imagined movement. *Nature Communications,**9*, Article 1617. 10.1038/s41467-018-03989-0 29691389 PMC5915435

[CR57] Knight, A. A., & Dagnall, P. R. (1967). Precision in Movements. *Ergonomics,**10*(3), 321–330. 10.1080/00140136708930874 6077520

[CR58] Kosslyn, S. M., Thompson, W. L., & Ganis, G. (2006). *The case for mental imagery*. Oxford University Press. 10.1093/acprof:oso/9780195179088.001.0001

[CR59] Kraeutner, S. N., Eppler, S. N., Stratas, A., & Boe, S. G. (2020). Generate, maintain, manipulate? Exploring the multidimensional nature of motor imagery. *Psychology of Sport and Exercise,**48*, Article 101673. 10.1016/j.psychsport.2020.101673

[CR60] Krüger, B., Hegele, M., & Rieger, M. (2022). The multisensory nature of human action imagery. *Psychological Research,**88*, 1870–1882. 10.1007/s00426-022-01771-y 36441293 PMC11315721

[CR61] Krüger, B., Zabicki, A., Grosse, L., Naumann, T., & Munzert, J. (2020). Sensory features of mental images in the framework of human actions. *Consciousness and Cognition,**83*, Article 102970. 10.1016/j.concog.2020.102970 32540626

[CR62] Kruger, J., & Dunning, D. (1999). Unskilled and unaware of it: How difficulties in recognizing one’s own incompetence lead to inflated self-assessments. *Journal of Personality and Social Psychology,**77*(6), 1121–1134. 10.1037/0022-3514.77.6.1121 10626367

[CR63] Lambert, K. J. M., Singhal, A., & Leung, A. W. S. (2024). The lateralized effects of Parkinson’s disease on motor imagery: Evidence from mental chronometry. *Brain and Cognition,**178*, Article 106181. 10.1016/j.bandc.2024.106181 38796902

[CR64] Lange, K., Kühn, S., & Filevich, E. (2015). “Just another tool for online studies” (JATOS): An easy solution for setup and management of web servers supporting online studies. *PLoS ONE,**10*(6), e0130834. 10.1371/journal.pone.0130834 26114751 PMC4482716

[CR65] Lo, S., & Andrews, S. (2015). To transform or not to transform: Using generalized linear mixed models to analyse reaction time data. *Frontiers in Psychology,**6*, 1171. 26300841 10.3389/fpsyg.2015.01171 PMC4528092

[CR66] López, N. D., Monge Pereira, E., Centeno, E. J., & Miangolarra Page, J. C. (2019). Motor imagery as a complementary technique for functional recovery after stroke: A systematic review. *Topics in Stroke Rehabilitation,**26*(8), 576–587. 10.1080/10749357.2019.1640000 31347992

[CR67] Louis, M., Collet, C., & Guillot, A. (2011). Differences in motor imagery times during aroused and relaxed conditions. *Journal of Cognitive Psychology,**23*(3), 374–382. 10.1080/20445911.2011.521739

[CR68] Macuga, K. L., Papailiou, A. P., & Frey, S. H. (2012). Motor imagery of tool use: Relationship to actual use and adherence to Fitts’ law across tasks. *Experimental Brain Research,**218*(2), 169–179. 10.1007/s00221-012-3004-0 22294026 PMC3351569

[CR69] Madan, C. R., & Singhal, A. (2013). Introducing TAMI: An objective test of ability in movement imagery. *Journal of Motor Behavior,**45*(2), 153–166. 10.1080/00222895.2013.763764 23557260

[CR70] Mandolesi, L., Passarello, N., & Lucidi, F. (2024). Differences in motor imagery abilities in active and sedentary individuals: New insights from backward-walking imagination. *Psychological Research,**88*(2), 499–508. 10.1007/s00426-023-01876-y 37773349 PMC10858124

[CR71] Martel, M., & Glover, S. (2023). TMS over dorsolateral prefrontal cortex affects the timing of motor imagery but not overt action: Further support for the motor-cognitive model. *Behavioural Brain Research,**437*, Article 114125. 10.1016/j.bbr.2022.114125 36167217

[CR72] Martin, K., Jacobs, S., & Frey, S. H. (2011). Handedness-dependent and -independent cerebral asymmetries in the anterior intraparietal sulcus and ventral premotor cortex during grasp planning. *NeuroImage,**57*(2), 502–512. 10.1016/j.neuroimage.2011.04.036 21554968 PMC3114104

[CR73] Martin, M. A., & Roberts, S. (2010). Jackknife-after-bootstrap regression influence diagnostics. *Journal of Nonparametric Statistics,**22*(2), 257–269. 10.1080/10485250903287906

[CR74] Maruff, P., Wilson, P. H., De Fazio, J., Cerritelli, B., Hedt, A., & Currie, J. (1999a). Asymmetries between dominant and non-dominant hands in real and imagined motor task performance. *Neuropsychologia,**37*(3), 379–384. 10.1016/S0028-3932(98)00064-5 10199649

[CR75] Maruff, P., Wilson, P., Trebilcock, M., & Currie, J. (1999b). Abnormalities of imagined motor sequences in children with developmental coordination disorder. *Neuropsychologia,**37*(11), 1317–1324. 10.1016/S0028-3932(99)00016-0 10530731

[CR76] Mathôt, S., Schreij, D., & Theeuwes, J. (2012). OpenSesame: An open-source, graphical experiment builder for the social sciences. *Behavior Research Methods,**44*(2), 314–324. 10.3758/s13428-011-0168-7 22083660 PMC3356517

[CR77] Moreno-Verdú, M., Hamoline, G., Van Caenegem, E. E., Waltzing, B. M., Forest, S., Valappil, A. C., … Khan, A. H. (2024). Guidelines for reporting action simulation studies (GRASS): Proposals to improve reporting of research in motor imagery and action observation. *Neuropsychologia*, *192*, Article 108733. 10.1016/j.neuropsychologia.2023.108733 37956956

[CR78] Moreno-Verdú, M., McAteer, S. M., Waltzing, B. M., Van Caenegem, E. E., & Hardwick, R. M. (2025). Development and validation of an open-source hand laterality judgement task for in-person and online studies. *Neuroscience,**572*, 93–107. 10.1016/j.neuroscience.2025.02.056 40064366

[CR79] Moreno-Verdú, M., Waltzing, B. M., Van Caenegem, E. E., Czilczer, C., Boidequin, L. F., Truong, C., ... Dahm, S. F. (2026). A comprehensive, open-source battery of movement imagery ability tests: Development and psychometric properties. *Behavior Research Methods,**58*(5), Article 120. 10.3758/s13428-026-03002-3 41981243

[CR80] Munzert, J., Lorey, B., & Zentgraf, K. (2009). Cognitive motor processes: The role of motor imagery in the study of motor representations. *Brain Research Reviews,**60*(2), 306–326. 10.1016/j.brainresrev.2008.12.024 19167426

[CR81] Muraki, E. J., & Pexman, P. M. (2021). Simulating semantics: Are individual differences in motor imagery related to sensorimotor effects in language processing? *Journal of Experimental Psychology: Learning, Memory, and Cognition,**47*(12), 1939–1957. 10.1037/xlm0001039 34197170

[CR82] Nederhof, A. J. (1985). Methods of coping with social desirability bias: A review. *European Journal of Social Psychology,**15*(3), 263–280. 10.1002/ejsp.2420150303

[CR83] Oishi, K., Kasai, T., & Maeshima, T. (2000). Autonomic response specificity during motor imagery. *Journal of Physiological Anthropology and Applied Human Science,**19*(6), 255–261. 10.2114/jpa.19.255 11204872

[CR84] Orliaguet, J.-P., & Coello, Y. (1998). Differences between actual and imagined putting movements in golf: A chronometric analysis. *International Journal of Sport Psychology,**29*(2), 157–169.

[CR85] Papaxanthis, C., Paizis, C., White, O., Pozzo, T., & Stucchi, N. (2012). The relation between geometry and time in mental actions. *PLoS ONE,**7*(11), Article e51191. 10.1371/journal.pone.0051191 23226487 PMC3511381

[CR86] Pathak, A., Patel, S., Karlinsky, A., Taravati, S., & Welsh, T. N. (2023). The “eye” in imagination: The role of eye movements in a reciprocal aiming task. *Behavioural Brain Research,**441*, Article 114261. 10.1016/j.bbr.2022.114261 36539164

[CR87] Plamondon, R., & Alimi, A. M. (1997). Speed/accuracy trade-offs in target-directed movements. *Behavioral and Brain Sciences,**20*(2), 279–303. 10.1017/S0140525X97001441 10096999

[CR88] Podda, J., Pedullà, L., Monti Bragadin, M., Piccardo, E., Battaglia, M. A., Brichetto, G., Bove, M., & Tacchino, A. (2020). Spatial constraints and cognitive fatigue affect motor imagery of walking in people with multiple sclerosis. *Scientific Reports*, *10*(1), Article 21938. 10.1038/s41598-020-79095-3 PMC7736576 33318605

[CR89] Pylyshyn, Z. W. (2002). Mental imagery: In search of a theory. *Behavioral and Brain Sciences,**25*(2), 157–182. 10.1017/S0140525X02000043 12744144

[CR90] Reed, C. L. (2002). Chronometric comparisons of imagery to action: Visualizing versus physically performing springboard dives. *Memory & Cognition,**30*(8), 1169–1178. 10.3758/BF03213400 12661849

[CR91] Rieger, M. (2012). Motor imagery in typing: Effects of typing style and action familiarity. *Psychonomic Bulletin & Review,**19*(1), 101–107. 10.3758/s13423-011-0178-6 22057418

[CR92] Rieger, M., Boe, S. G., Ingram, T. G. J., Bart, V. K. E., & Dahm, S. F. (2024). A theoretical perspective on action consequences in action imagery: Internal prediction as an essential mechanism to detect errors. *Psychological Research,**88*(6), 1849–1858. 10.1007/s00426-023-01812-0 36961546 PMC7616356

[CR93] Rieger, M., Dahm, S. F., & Koch, I. (2017). Inhibition in motor imagery: A novel action mode switching paradigm. *Psychonomic Bulletin & Review,**24*(2), 459–466. 10.3758/s13423-016-1095-5 27363713 PMC5120687

[CR94] Roberts, R., Callow, N., Hardy, L., Markland, D., & Bringer, J. (2008). Movement imagery ability: Development and assessment of a revised version of the Vividness of Movement Imagery Questionnaire. *Journal of Sport & Exercise Psychology,**30*(2), 200–221. 10.1123/jsep.30.2.200 18490791

[CR95] Roberts, J., Owen, R., & Wakefield, C. (2025). Mental chronometry: Do imagined times merely relate to task duration? *Journal of Motor Behavior*. 10.1080/00222895.2025.2525907 40622302

[CR96] Robin, N., Dominique, L., Toussaint, L., Blandin, Y., Guillot, A., & Her, M. L. (2007). Effects of motor imagery training on service return accuracy in tennis: The role of imagery ability. *International Journal of Sport and Exercise Psychology,**5*(2), 175–186. 10.1080/1612197X.2007.9671818

[CR97] Ross, L., Greene, D., & House, P. (1977). The “false consensus effect”: An egocentric bias in social perception and attribution processes. *Journal of Experimental Social Psychology,**13*(3), 279–301. 10.1016/0022-1031(77)90049-X

[CR98] Ryu, E. (2011). Effects of skewness and kurtosis on normal-theory based maximum likelihood test statistic in multilevel structural equation modeling. *Behavior Research Methods,**43*(4), 1066–1074. 10.3758/s13428-011-0115-7 21671139

[CR99] Schott, N. (2013). Test zur Kontrollierbarkeit der Bewegungsvorstellungsfähigkeit (TKBV) bei älteren Erwachsenen. *Zeitschrift für Gerontologie und Geriatrie,**46*(7), 663–672. 10.1007/s00391-013-0520-x 23912128

[CR100] Schwarzkopf, D. S. (2024). What is the true range of mental imagery? *Cortex; A Journal Devoted to the Study of the Nervous System and Behavior,**170*, 21–25. 10.1016/j.cortex.2023.09.013 37949779

[CR101] Simonsmeier, B. A., Andronie, M., Buecker, S., & Frank, C. (2021). The effects of imagery interventions in sports: A meta-analysis. *International Review of Sport and Exercise Psychology,**14*(1), 186–207. 10.1080/1750984X.2020.1780627

[CR102] Sirigu, A., Cohen, L., Duhamel, J. R., Pillon, B., Dubois, B., Agid, Y., & Pierrot-Deseilligny, C. (1995). Congruent unilateral impairments for real and imagined hand movements. *NeuroReport,**6*(7), 997–1001. 10.1097/00001756-199505090-00012 7632907

[CR103] Smith, D., & Wakefield, C. (2013). A timely review of a key aspect of motor imagery: A commentary on Guillot et al. (2012). *Frontiers in Human Neuroscience,**7*, Article 761. 10.3389/fnhum.2013.00761 24265614 PMC3821274

[CR104] Smits-Engelsman, B. C. M., & Wilson, P. H. (2013). Age-related changes in motor imagery from early childhood to adulthood: Probing the internal representation of speed-accuracy trade-offs. *Human Movement Science,**32*(5), 1151–1162. 10.1016/j.humov.2012.06.006 23164627

[CR105] Suggate, S. P. (2024). Beyond self-report: Measuring visual, auditory, and tactile mental imagery using a mental comparison task. *Behavior Research Methods,**56*(8), 8658–8676. 10.3758/s13428-024-02496-z 39271632 PMC11525388

[CR106] Van Caenegem, E. E., Moreno-Verdú, M., Waltzing, B. M., Hamoline, G., Lennart, F., & Hardwick, R. M. (2026). Greater neural overlap between motor imagery and working memory than with movement execution: A meta-analytic comparison. *Imaging Neuroscience,**4*, Article IMAG.a.1095. 10.1162/IMAG.a.1095 41537051 PMC12797143

[CR107] Viviani, P., & Terzuolo, C. (1982). Trajectory determines movement dynamics. *Neuroscience,**7*(2), 431–437. 10.1016/0306-4522(82)90277-9 7078732

[CR108] Watt, A. (2003). *Development and validation of the Sport Imagery Ability Measure* [Doctoral Dissertation, Victoria University of Technology]. VU Research Repository. https://vuir.vu.edu.au/16135/1/WATT_2003compressed.pdf

[CR109] Williams, S. E., Cumming, J., Ntoumanis, N., Nordin-Bates, S. M., Ramsey, R., & Hall, C. (2012). Further validation and development of the movement imagery questionnaire. *Journal of Sport and Exercise Psychology,**34*(5), 621–646. 10.1123/jsep.34.5.621 23027231

[CR110] Williams, S. E., Guillot, A., Di Rienzo, F., & Cumming, J. (2015). Comparing self-report and mental chronometry measures of motor imagery ability. *European Journal of Sport Science,**15*(8), 703–711. 10.1080/17461391.2015.1051133 26313631

[CR111] Wilson, P. H., Maruff, P., Ives, S., & Currie, J. (2001). Abnormalities of motor and praxis imagery in children with DCD. *Human Movement Science,**20*(1–2), 135–159. 10.1016/S0167-9457(01)00032-X 11471394

[CR112] Wolpert, D. M., & Ghahramani, Z. (2000). Computational principles of movement neuroscience. *Nature Neuroscience,**3*(11), 1212–1217. 10.1038/81497 11127840

[CR113] Wong, L., Manson, G. A., Tremblay, L., & Welsh, T. N. (2013). On the relationship between the execution, perception, and imagination of action. *Behavioural Brain Research,**257*, 242–252. 10.1016/j.bbr.2013.09.045 24100120

[CR114] Worringham, C. J., & Beringer, D. B. (1998). Directional stimulus-response compatibility: A test of three alternative principles. *Ergonomics,**41*(6), 864–880. 10.1080/001401398186694 9629069

[CR115] Wright, D., Scott, M., Kraeutner, S., Barhoun, P., Bertollo, M., Campbell, M., Waltzing, B., Dahm, S., Esselaar, M., Frank, C., Hardwick, R., Fuelscher, I., Marshall, B., Hodges, N., Hyde, C., & Holmes, P. (2024). An international estimate of the prevalence of differing visual imagery abilities. *Frontiers in Psychology,**15*, Article 1454107. 10.3389/fpsyg.2024.1454107 39474086 PMC11518826

[CR116] Wu, J., Yang, J., & Honda, T. (2010). Fitts’ law holds for pointing movements under conditions of restricted visual feedback. *Human Movement Science,**29*(6), 882–892. 10.1016/j.humov.2010.03.009 20659774

[CR117] Yoxon, E., Tremblay, L., & Welsh, T. N. (2015). Effect of task-specific execution on accuracy of imagined aiming movements. *Neuroscience Letters,**585*, 72–76. 10.1016/j.neulet.2014.11.021 25445380

