# Supplementary material for: One size Fitts’ all? Reconsidering the use of deviations from real-time action imagery in mental chronometry tasks
Source: Behav Res Methods. 2026 Jul 30;58(9):254. doi: 10.3758/s13428-026-03124-8 (PMC13424699; doi:10.3758/s13428-026-03124-8)
Supplement: Supplementary file 1 — Supplementary file1 (PDF 291 kb) [file 13428_2026_3124_MOESM1_ESM.pdf]

Supplementary Material

Fig. A Participants' mean movement time in ms by CRFT condition and movement difficulty

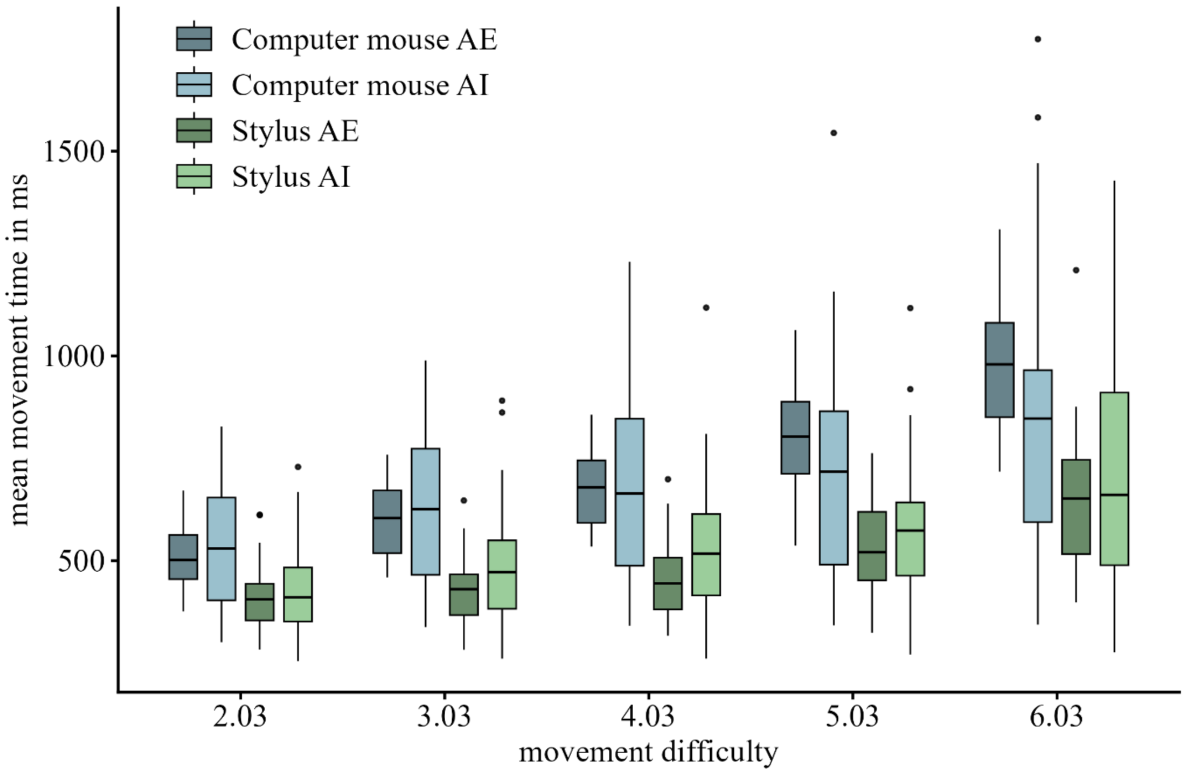

Table A Comparison of sequentially fitted overall GLMMs

							LR test[a]	
Model	Fixed effects	Random effects over particpants	AIC	BIC	LL	#p	df	Λ
M1	Intercept	Intercept	117199	117220	-58597	3	2	
M2	"	+ Movement difficulty[b]	113986	114021	-56988	5	1	3217.60***
M3	+ Movement difficulty	"	113946	113989	-56967	6	1	41.23***
M4	+ Tool	"	111022	111072	-55504	7	1	2926.13***
M5	+ Action	"	111017	111074	-55501	8	1	6.98**
M6	+ Tool × movement difficulty	"	110747	110810	-55364	9	1	272.56***
M7	+ Tool × action	"	110577	110648	-55278	10	1	171.64***
M8	+ Action × movement difficulty	"	110499	110577	-55239	11	1	79.92***
**M9**	**+ Action × tool × movement difficulty**	**"**	**110358**	**110442**	**-55167**	**12**	**1**	**143.55*****

Simpler model nested in more complex model (e.g., M1 nested in M2, M2 nested in M3)

LL = log-likelihood. #p = *n* parameters

Best fitting model printed in bold

[a] Likelihood ratio test between the model and nested model

[b] Random slopes

*** $p < .001$, ** $p < .01$, * $p < .05$

Table B Sensitivity analysis comparing results of the final overall GLMM with a GLMM additionally including experience with action imagery

	Fixed effects					
	b_{M1}	SE_{M1}	p_{M1}	b_{M2}	SE_{M2}	p_{M2}
Intercept	631	5	< .001	633	4	< .001
Movement difficulty	78	4	< .001	78	4	< .001
Tool	174	3	< .001	174	3	< .001
Action	28	2	< .001	28	3	< .001
Movement difficulty × tool	31	2	< .001	31	2	< .001
Tool × action	94	4	< .001	94	5	< .001
Movement difficulty × action	24	2	< .001	24	2	< .001
Movement difficulty × tool × action	45	3	< .001	45	3	< .001
Experience				36	4	< .001
	Random effects					
	Var	*SD*	*r*	*Var*	*SD*	*r*
Participant (intercept)	1840	43		1834	43	
Movement difficulty (slope)	217	14	.60	216	15	.61

Movement time as dependent variable
Participant as grouping variable
M1 = final overall model, M2 = M1 + experience
Movement difficulty = [–2; 2]
Tool: –0.5 = stylus, 0.5 = computer mouse
Action: –0.5 = action imagery, 0.5 = action execution
Experience: –0.5 = no experience with action imagery, 0.5 = experience with action imagery through sports or previous experiments

A sensitivity analysis (Table B) was conducted by comparing the final overall model with a model that additionally included a fixed effect for experience (dichotomous single-item indicating prior experience with action imagery through sports or previous experiments). Model comparison showed that the inclusion of experience did not significantly improve model fit ($\Delta\chi^2(1) = 0.96$, $p = .33$; $AIC_{M1} = 110358$, $AIC_{M2} = 110359$; $BIC_{M1} = 110442$, $BIC_{M2} = 110451$). Experience was associated with significantly slower movement times. Inclusion of experience did not meaningfully alter the estimates for any fixed or random effect, indicating that the overall pattern of results remained stable.

Table C Random effects for post hoc GLMMs testing differences of movement times in ms and slopes *b* between CRFT conditions

Post hoc models	*Var*	*SD*	*r*
1: Stylus AE vs. stylus AI			
Participant (intercept)	1874	43	
Movement difficulty (slope)	217	15	.56
2: Computer mouse AE vs. computer mouse AI			
Participant (intercept)	3315	58	
Movement difficulty (slope)	382	20	.64
3: Computer mouse AE vs. stylus AE			
Participant (intercept)	1154	34	
Movement difficulty (slope)	146	12	.53
4: Computer mouse AI vs. stylus AI			
Participant (intercept)	4056	64	
Movement difficulty (slope)	483	22	.65

Participant as grouping variable

Movement difficulty = [–2; 2]

AE = action execution

AI = action imagery

Table D Comparison of GLMMs explaining movement time per CRFT condition by movement difficulty vs. target width

Model	Nested	Fixed effects	Random effects over particpants	AIC	BIC	LL	#p
Stylus AE							
M1a		Intercept + movement difficulty	Intercept	25098	25120	-12545	4
**M1b**	**M1a**	**“**	**+ Movement difficulty[a]**	**24550**	**24584**	**-12269**	**6**
M2a		Intercept + width	Intercept	25699	25722	-12846	4
M2b	M2a	“	+ Width[a]	26784	26818	-13386	6
Stylus AI							
M1a		Intercept + movement difficulty	Intercept	26703	26726	-13348	4
**M1b**	**M1a**	**“**	**+ Movement difficulty[a]**	**25783**	**25817**	**-12886**	**6**
M2a		Intercept + width	Intercept	26935	26958	-13464	4
M2b	M2a	“	+ Width[a]	26293	26327	-13140	6
Computer mouse AE							
M1a		Intercept + movement difficulty	Intercept	26956	26979	-13474	4
**M1b**	**M1a**	**“**	**+ Movement difficulty[a]**	**26767**	**26801**	**-13378**	**6**
M2a		Intercept + width	Intercept	27623	27646	-13808	4
M2b	M2a	“	+ Width[a]	28121	28156	-14055	6
Computer mouse AI							
M1a		Intercept + movement difficulty	Intercept	27750	27773	-13871	4
**M1b**	**M1a**	**“**	**+ Movement difficulty[a]**	**26956**	**26990**	**-13472**	**6**
M2a		Intercept + width	Intercept	27907	27929	-13949	4
M2b	M2a	“	+ Width[a]	30021	30056	-15005	6

LL = log-likelihood, #p = *n* parameters, AE = action execution, AI = action imagery

Best fitting models printed in bold

Singular models printed in gray

[a] Random slopes

Table E Spearman correlations between absolute deviation, relative deviation, constraint and Fitts' law AIA scores in both tool conditions

Item	Absolute deviation		Relative deviation		Constraint		Fitts' law	
	Stylus	Computer mouse	Stylus	Computer mouse	Stylus	Computer mouse	Stylus	Computer mouse
Absolute deviation								
Stylus	—							
Computer mouse	.02	—						
Relative deviation								
Stylus	**.96**	.04	—					
Computer mouse	.00	**.98**	.05	—				
Constraint								
Stylus	-.14	.19	-.08	.18	—			
Computer mouse	.18	**.40**	.16	**.37**	.05	—		
Fitts' law								
Stylus	.03	-.13	.15	-.09	.10	-.04	—	
Computer mouse	-.11	.04	-.06	.07	-.01	**.43**	**.51**	—

Significant values printed in bold

Absolute and relative deviation scores correlated very strongly with each other within each tool but did not correlate across tools (Table E). For the computer mouse, but not the stylus, the constraint approach showed moderate correlations with the other three mental chronometry approaches. This pattern suggests that deviation-based mental chronometry approaches largely capture task- and tool-specific variance rather than the underlying construct of action imagery ability.

Table F Correlations between absolute deviation, relative deviation, constraint, Fitts' law AIA scores and action imagery items in both tool conditions

Item	Absolute deviation		Relative deviation		Constraint		Fitts' law	
	Stylus[a]	Computer mouse[b]	Stylus[a]	Computer mouse[b]	Stylus[a]	Computer mouse[b]	Stylus[b]	Computer mouse[b]
Stylus AI								
Ease	.29	.08	.27	.04	-.26	.07	**-.48**	.00
Speed	.17	-.25	.20	-.21	-.06	-.11	-.22	.05
Maintenance	-.09	.04	-.10	.05	-.28	.07	-.13	.13
Control	-.01	.16	-.02	.15	-.31	.00	-.12	.20
Clarity	.25	.07	.29	.06	-.31	.09	-.15	.10
Visual	.09	**.37**	.10	**.38**	-.09	**.39**	-.27	.17
Kinesthetic	.30	.06	**.39**	.07	-.15	.16	-.10	.10
Tactile	-.16	-.01	-.14	.00	-.30	.05	.00	.06
Auditory	.09	.08	.17	.03	.04	.09	.13	.06
Computer mouse AI								
Ease	**.38**	.10	.33	.06	-.15	.16	**-.45**	.05
Speed	.29	-.10	.31	-.09	-.01	.07	-.11	-.07
Maintenance	.21	.05	.12	.04	-.11	-.23	**-.49**	-.26
Control	.36	.30	**.37**	.26	.00	-.02	**-.42**	-.09
Clarity	**.37**	.23	.34	.19	.11	-.15	**-.40**	-.29
Visual	.30	.33	.25	.28	.15	**.51**	-.32	.05
Kinesthetic	.27	.15	.28	.10	-.02	-.10	**-.46**	-.27
Tactile	.02	.13	-.05	.13	-.29	-.11	-.25	-.07
Auditory	.18	.12	.22	.08	-.14	-.10	.06	.13

Significant values printed in bold

[a] Spearman correlation

[b] Pearson correlation
